# Can We Extract Physics-like Energies from Generative Protein Diffusion Models?

**DOI:** 10.1101/2025.11.28.690021

**Published:** 2025-12-17

**Authors:** Sudeep Sarma, Harrison Truscott, Da Xu, Kendall Reid, Lee-Shin Chu, Jacky Chen, Jeffrey J. Gray

**Affiliations:** 1 Department of Chemical and Biomolecular Engineering, Johns Hopkins University, Baltimore, MD 21218, USA; 2 Bioinformatics Program, Center for Biotechnology Education, Johns Hopkins University, Baltimore, MD 21218, USA; 3 CMU-Pitt Computational Biology, Dept. of Computational & Systems Biology, University of Pittsburgh, Pittsburgh, PA 15260, USA

## Abstract

Diffusion models have emerged as the state-of-the-art method in generative artificial intelligence (AI) and have shown great success in image synthesis, video generation, molecular design, and protein structure prediction. For biophysical problems, such as protein folding and association, a fundamental question in diffusion-based methods is how their learned functions correspond to thermodynamics. In this paper, we study diffusion models through the lens of theoretical biophysics, analyzing their underlying formulation of potentials and exploring their applications in scoring protein interactions. We develop simple theories rooted in statistical physics that relate thermodynamic potentials to the negative log of the probability of observing a system in a particular state. We include dimensional analysis of diffusion model equations and a table mapping AI and physics jargon. We then test a diffusion model’s ability to capture learned energies as negative log-likelihood values, $-log\;p_{0}\left(x_{0}\right)$, by integrating over the diffusion-generated path or a probability flow path. We test these integrals on a simple 1D Gaussian mixture diffusion model and a protein-docking diffusion model, DFMDock. In the 1D case, we find that integration over both diffusion and flow paths can accurately recover ground truth probabilities. When we extract the learned docking energies for cases where DFMDock succeeds, we observe energy funnels with the minimum energy near the experimental docked structure, like those we observe with Rosetta, an empirically tuned physics-based biomolecular modeling suite. The learned energy performs comparably or outperforms Rosetta interface energy in 6 out of 25 cases at ranking the correctness of docked poses. These data show that we can extract a relevant learned energy function from a diffusion model and compare it to physical energy functions.

## I. INTRODUCTION

Diffusion models are a class of deep-generative models that have shown great promise in image [1–3] and video generation [4], protein design [5, 6], and protein structure prediction [7–9]. Diffusion models were originally formulated by Sohl-Dickstein *et al.* [10] in analogy with nonequilibrium thermodynamics. They work in two stages. In forward diffusion, a data distribution is transformed to a prior (typically a Gaussian) over a series of time steps to learn an effective force field (the score), which is the time-dependent gradient of the logarithmic probability density function [11]. The score is learned by a deep neural network matching the noise added in training. One can then use the learned score to sample the underlying probability distribution of the data during a backward diffusion process starting from the prior. Leading image generation tools such as DALL-E [1], Stable Diffusion [2], and Midjourney [3] and text-to-video models like Sora [4] are all based on diffusion models. In protein design, breakthroughs such as RFDiffusion [5] and Chroma [6] have led to novel biomolecules that fold and bind in the lab. RFDiffusion represents amino acid residues as rigid frames and generates protein backbones from an ideal-gas-like prior. Chroma diffuses in polymer backbone torsion space, respecting conformational statistics of polymer ensembles.

In 2024, Deepmind released AlphaFold3 (AF3) [7], replacing AlphaFold2’s [12] structure module with a diffusion module capable of predicting structures of proteins, protein complexes and post-translational modifications. AF3 drew from previous pioneering studies of diffusion for biomolecules [5, 6, 13], and has since inspired several derivative models (Chai-1 [8], Boltz-2 [14], and OpenFold3 [15]). The incredible success of AF3 and other protein deep learning models is in some ways surprising because these methods outperform physics-based methods like Rosetta [16, 17], which is based on decades of research into conformational sampling and scoring that follow energy functions derived from or inspired by physical principles. It raises the question of whether these high-performing deep-learning methods have implicitly learned a potential akin to nature’s free energy function.

The generative model training objective is to learn to sample from a distribution that matches known protein structures. In other words, the sampling probability should be maximized at real protein structures, similar to Anfinsen’s dogma [18], which states that protein structures are at their lowest free energy state (i.e., in equilibrium). There are many functional forms that can have minima at the same (folded) states, so an AI protein model’s learned energy function does not need to match nature’s energy function. Additionally, diffusion models do not typically allow direct access to the probability of a given state. Here, we seek general approaches to extract information from a diffusion model in the form of its learned potential. In this way, we can compare this potential to physics-based energy functions and consider how AI and physics-based methods might be combined or improved.

To better understand the inherent potentials that diffusion models learn, we focus on a tractable and interpretable model for the protein-protein docking problem [19]. Previously, we introduced DFMDock (Denoising Force Matching Dock), a diffusion model for rigid-body protein-protein docking [20]. It is trained by adding translational and rotational noise to experimentally determined, bound protein complexes, which the model then learns to reverse through a denoising force-matching objective. During inference, the model inputs two unbound monomers and generates the structure of the bound complex. For our model study, we use a DFMDock version trained only on the translation docking space. In this way, we can also make comparison with a 1D translational diffusion model of a Gaussian mixture.

We formulate diffusion models in the language of statistical thermodynamics and explore how protein diffusion models like DFMDock score biomolecular structures and complexes. Statistical thermodynamics provides a framework for predicting the static and dynamic properties of a many-body system from its microscopic constituents and their interactions [21]. For systems in equilibrium, the Boltzmann distribution relates the probability of finding a system in a particular configuration x to its energy E(x) as:

(1) $$p(x)=\frac{e^{-\beta E(x)}}{Z},$$

where β is the system’s inverse temperature and the partition function Z is integrated over the space of all possible system configurations:

(2) $$Z=\int_{\Omega }e^{-\beta E(x)}dx.$$

We assess a diffusion model’s ability to capture energies by introducing a framework to extract negative log-likelihood values (NLL) of a sample $x_{0}$ at diffusion time t=0, $-log\;p_{0}\left(x_{0}\right)$. We establish a relationship between the learned energy, $-log\;p_{0}\left(x_{0}\right)$, and the thermodynamic free energy of the configuration, $E_{0}\left(x_{0}\right)$.

**FIG. 1.**
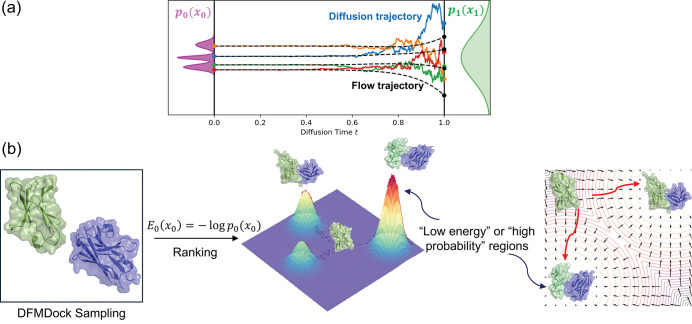
(a) Diffusion (SDE paths, solid colors) and flow (ODE paths, dashed black) trajectories from a noise (prior) distribution, $p_{1}\left(x_{1}\right)$, at time t=1 to the data distribution, $p_{0}\left(x_{0}\right)$, at time t=0. (b) Negative log-likelihood values of docked protein complexes are a measure of learned protein-protein interaction energy.

This relationship connects deep learning models with physics by mapping learned potentials with fundamental thermodynamic quantities. We explore multiple approaches to calculating $E_{0}\left(x_{0}\right)$ by integrating the implicitly learned energy gradient using $\nabla_{x}\;log\;p$ over both noisy diffusion paths and the smooth deterministic path generated by the probability flow ordinary differential equation (ODE) as described in [11]. As a test for our approach, we train a simple 1D diffusion model to learn a Gaussian mixture and compare generated samples’ $-log\;p_{0}\left(x_{0}\right)$ values using our integral methods to the ground truth values. We then apply the same approach to a protein docking diffusion system and compare the learned energies with Rosetta energies (Fig. 1).

To our knowledge this is the first attempt at extracting potentials from a biomolecular diffusion model and comparing with a physical energy function. This is also the first exploration of using paths other than the flow-equivalent ODE for likelihood computation. We aspire to improve the interpretability of deep-learning models for protein-protein interactions and open the door to building more robust physics informed tools for biomolecular engineering.

## II. RELATED WORK

Protein-protein interactions (PPIs) drive a wide range of biological and chemical processes. They are involved in most cellular functions in living organisms, such as signaling, regulation, and recognition. Understanding their three-dimensional structures provides atomic-level insight into the mechanisms of these functions [22]. Traditional protein docking methods use sampling algorithms such as local-shape matching and Monte Carlo search algorithms to generate plausible docked conformations [23, 24]. Scoring functions, such as the Rosetta interface score function, can then be used to evaluate the physical energy of these docked conformations [25]. However, these search and scoring protocols are computationally intensive. Recently, new generative deep learning methods, especially diffusion models, have shown promise in addressing the protein-docking problem.

The first diffusion model applied to molecular docking, DiffDock, learned small-molecule–protein docking with denoising score matching on the translation, rotation, and torsion spaces of small molecules [13]. The developers subsequently introduced DiffDock-PP, a diffusion model for rigid protein docking [26] that learned to translate and rotate unbound protein structures to their bound conformations. DiffMaSIF is a diffusion framework for protein docking that uses an encoder-decoder architecture to learn physical surface patch complementarity [27]. LatentDock first trains a variational autoencoder on protein sequences and structures and then diffuses in the latent space to produce the final conformations of the protein complex [28].

Like diffusion models, energy-based models (EBMs) are a class of generative models that have been applied in modeling protein-protein interactions. EBMs are trained to learn a scalar energy function over the training data distribution, where lower energy values correspond to higher probability [29]. DSMBind is an EBM whose energy function is optimized by matching its gradient to that of the forward diffusion process to predict binding energies of protein-protein interactions [30]. DockGame learned an energy function via supervision from physics-based models and self-supervision via score-matching with diffusion models for rigid multimeric protein docking [31]. EBMDock uses an energy-based learning framework and Langevin dynamics sampling for docking pose prediction [32]. Borisiak *et al.* trained an energy-based diffusion network to score mutations on peptides and CDR3 loops within TCR-pMHC interfaces [33]. Plainer *et al.* trained an energy-based model with an additional Fokker-Planck based loss on the model’s temporal gradient to improve the consistency of the energy function at time t=0. They showed that the gradient of this improved energy can be used as a force function for coarse-grained molecular dynamics simulations [34].

These models have shown promising empirical results. Other recent work has focused on the theoretical under-pinnings of diffusion models. Diffusion models derive from non-equilibrium physics [10], but several papers have also conceptualized diffusion models using tools of equilibrium statistical mechanics. Ambrogioni *et al.* [35] showed that generative diffusion models undergo second-order phase transitions as described by mean-field theory. Sclocchi *et al.* [36] found that during the backward diffusion process, the system undergoes a phase transition such that the probability of reconstructing high-level features like the class of an image suddenly drops. Biroli *et al.* [37] showed that the backward diffusion process has three dynamical regimes, and they characterized the cross-overs between them as speciation (analogous to symmetry-breaking phenomena) and collapse (analogous to glass transition).

The principle of extracting learned likelihood from score-based generative diffusion models was first introduced by Song *et al.* (2020) [11] using their probability flow ODE. By converting the diffusion process into an equivalent process using stochasticity-free ODEs, they could use the instantaneous change-of-variables formula from [38] to integrate the probability change along a flow ODE trajectory. By contrast, we formulate likelihood recovery in terms of the Fokker-Planck equation describing the diffusion process. The instantaneous change-of-variables formula is a special case of this Fokker-Planck formulation when integrated over the flow trajectory, thanks to the equivalence between the ODE and diffusion process [11, 38], meaning our formulation generalizes the original likelihood formula to any (piecewise-differentiable) path.

Our paper focuses on devising general approaches to extract the learned likelihood function from diffusion models, and we compare the learned energy with physical energy potentials for protein docking, thus contributing to the interpretability of diffusion models in protein structure prediction and design.

## III. THEORY

### A. Stochastic Differential Equations (SDEs) in Diffusion Models

The goal of generative modeling is to learn to transform a distribution of noise into samples that closely resemble a particular data distribution, such as the distribution of naturally occurring protein structures. In diffusion modeling, we first describe a transformation from noise to data—the *forward* diffusion process—and train a neural network to undo this transformation during the *reverse* diffusion process.

The forward diffusion process is obtained by incrementally noising a distribution of samples, $p_{0}$, until the samples converge to a known prior distribution, $p_{1}$. To describe the transformation of data to noise, we introduce the continuous variable t∈[0,1], which indexes a continuum of probability distributions smoothly interpolating from $p_{0}$ to $p_{1}$.

The variable t is referred to as “time” in the machine learning literature, but for a protein or other physical system, it actually represents an alchemical transformation. For example, RFDiffusion diffuses protein structures into an oriented, ordered ideal gas. We can draw parallels with alchemical free energy methods that gradually change one molecule into another along a transformation variable, allowing the calculation of the excess chemical potential through a nonphysical perturbation [39, 40]. To clarify the jargon between the AI and physical chemistry communities, we summarize the interpretation of the diffusion model terms in Table I, where we differentiate the alchemical time dimension [τ] from the physical time dimension [T].

Mathematically, the incremental noising is formulated with a stochastic differential equation (SDE) known as the forward SDE:

(3) $$dx_{t}=f\left(x_{t},t\right)dt+g(t)dw_{t}.$$

In the forward diffusion process, a given point $x_{0}\in R^{n}$ sampled from the data distribution, $x_{0}∼p_{0}$, follows this SDE from time t=0 to t=1, arriving at some destination $x_{1}$ in the prior distribution, $x_{1}∼p_{1}$ [11]. The process is parameterized by two functions: the drift function, $f\left(x_{t},t\right)\;:R^{n}\times R\to R^{n}$, and the diffusion coefficient, $g\left(t\right):R\to R$. $w_{t}$ is a Wiener process describing stochastic Brownian motion, where $dw_{t}$ is an infinitesimal random perturbation sampled at each time t from a Gaussian with mean 0 and variance dt.

The forward SDE is constructed with specific choices f and g so that the resulting *marginal* probability distribution of this process, $p\left(x_{t},t\right)$-the probability of observing a point $x_{t}$ at time t, marginalized over all initial points $x_{0}$ and stochastic trajectories from $x_{0}$ to $x_{t}$—converges to the desired prior distribution $p_{1}$ at t=1. By definition, $p\left(x_{0},0\right)$ also matches our data distribution $p_{0}\left(x_{0}\right)$. Moreover, $p\left(x_{t},t\right)$ defines a continuum of distributions p(⋅,t), smoothly interpolating from $p_{0}$ to $p_{1}$.

In our 1D diffusion model and DFMDock, we set $f\left(x_{t},t\right)=0$ and $g(t)=\sigma_{0}{\left(\frac{\sigma_{1}}{\sigma_{0}}\right)}^{t}$; for our 1D diffusion model, $\sigma_{0}=0.1$ and $\sigma_{1}=70$, and for DFMDock, $\sigma_{0}=0.1$ and $\sigma_{1}=30$. In the language of [11], setting $f\left(x_{t},t\right)=0$ means our diffusion process is “variance exploding.” In DFMDock, $dw_{t}$ represents random translations of the ligand protein.

During inference, we use the reverse SDE [41]:

(4) $$dx_{t}=[f\left(x_{t},t\right)-g(t{)}^{2}\nabla_{x}log\;p\left(x_{t},t\right)]dt+g\left(t\right)d{\overset{‾}{w}}_{t},$$

where time flows backwards from 1 to 0, dt is an infinitesimal negative timestep and ${\overset{‾}{w}}_{t}$ is a reverse-time Wiener process. This SDE models the reverse diffusion process: starting from t=1 and a sample $x_{1}∼p_{1}$, the marginal distribution of the reverse SDE transforms the prior distribution $p_{1}$ into the data distribution $p_{0}$ as t *decreases* from 1 to 0. $\nabla_{x}log\;p\left(x_{t},t\right)$, known as the *score*, is the spatial gradient of the log of the marginal probability of the process at time t.

By construction, this reverse SDE has an identical marginal distribution to the forward SDE [41]. The only unknown in eq. (4) is the score, so in score-based generative modeling, the diffusion model consists of a neural network, $s_{\theta }\left(x_{t},t\right)$, parameterized by weights θ and trained to approximate the score given a sample $x_{t}$ and a time t (potentially conditioned on other aspects of the process, such as protein sequence identity). Since the marginal probability $p\left(x_{0},0\right)$ of arriving at a point $x_{0}$ via the reverse SDE should match the data distribution $p_{0}\left(x_{0}\right)$, sampling a point $x_{1}$ from the prior and following the reverse SDE using the learned score should produce samples $x_{0}$ with the same probability as sampling from the data distribution directly.

The marginal distribution is generally intractable to solve at training time, as we only have a finite dataset of samples from $p_{0}$ and thus cannot marginalize over the entire distribution. However, we can still evaluate the conditional probability $q\left(x_{t},t|x_{0}\right)$ of reaching a point $x_{t}$ at time t when starting from a single point in the data distribution, $x_{0}$, instead of the entire $p_{0}$ distribution. Vincent (2011) [42] proved that matching the learned score with q conditioned on each of the data points in turn will converge to the marginal distribution as the size of the dataset grows. This results in the so-called denoising score matching objective that is used to train score-based diffusion models:

(5) $$ℒ\left(\theta ,t\right)=E_{q\left(x_{t},t,x_{0}\right)}\left[\frac{1}{2}{\left\|s_{\theta }\left(x_{t},t\right)-\nabla_{x}\log q\left(x_{t},t|x_{0}\right)\right\|}_{2}^{2}\right],$$

where the expectation is evaluated as a weighted sum over samples from the forward diffusion process starting at the points in the training dataset. In this way, score-based diffusion models implicitly incorporate information about all possible diffusion trajectories into the score. We reason that it should therefore be possible to recover a trained model’s understanding of the ground-truth probability distribution—and thus, its understanding of the ground truth *energy*—without needing to explicitly marginalize over all paths through diffusion space.

### B. Forces and Energies in Diffusion Models

In the case of protein docking or folding, our data distribution $p_{0}$ is assumed to sample from the distribution of system states at thermal equilibrium, corresponding to stably folded or docked proteins. Thus, we can define an energy $E_{0}\left(x_{0}\right)$ for any equilibrium state $x_{0}$ based on $p_{0}$:

(6) $$p_{0}\left(x_{0}\right)=\frac{e^{-\beta E_{0}\left(x_{0}\right)}}{Z_{0}},$$

where $Z_{0}$ is the partition function associated with the system at equilibrium, calculated by integrating over the space of all system states:

(7) $$Z_{0}=\int_{\Omega }e^{-\beta E_{0}\left(x_{0}\right)}dx_{0},$$

and β corresponds to the system’s inverse-temperature, $\beta =\frac{1}{kT}$.

Diffusion models are trained to allow sampling from $p_{0}$, meaning they must have an implicit understanding of this energy function at equilibrium. However, they cannot output $p_{0}\left(x_{0}\right)$ directly. Instead, as discussed earlier, score-based diffusion models learn the *score* of the marginal distribution $p\left(x_{t},t\right)$ of the diffusion process:

(8) $$s_{\theta }\left(x_{t},t\right)\approx \nabla_{x}\log p\left(x_{t},t\right),$$

where $s_{\theta }\left(x_{t},t\right)$ is a neural network parameterized by weights θ.

We can, however, analyze what this score means in terms of our energy formulation. If we also assume that the system of states $x_{t}$ described by $p\left(x_{t},t\right)$ at constant time t is at pseudo-equilibrium (in other words, the diffusion process is quasi-static), we can define an energy $E_{t}$ for an arbitrary time t in the diffusion process by generalizing eq. (6):

(9) $$p\left(x_{t},t\right)=\frac{e^{-\beta_{t}E_{t}\left(x_{t}\right)}}{Z_{t}},$$

where $\beta_{t}$ and $Z_{t}$ are the inverse temperature and partition functions respectively at time t. Since we are defining this energy based on the probability alone, however, we are free to choose $\beta_{t}$ and $Z_{t}$, defining the scaling and reference energy of $E_{t}$ respectively. For purposes of this paper we thus assume a constant $\beta_{t}=\beta$ and $Z_{t}=Z_{0}=Z$:

(10) $$p\left(x_{t},t\right)=\frac{e^{-\beta E_{t}\left(x_{t}\right)}}{Z}.$$

Taking the log of both sides isolates the energy term (with a coefficient of -β):

(11) $$\log p\left(x_{t},t\right)=-\beta E_{t}\left(x_{t}\right)-\log Z.$$

And since Z is independent of $x_{t}$, taking the spatial gradient of both sides yields the score solely in terms of energy:

(12) $$\nabla_{x}\log p\left(x_{t},t\right)=-\beta \nabla_{x}E_{t}\left(x_{t}\right).$$

In classical physics, the negative gradient of energy with respect to position, $-\nabla_{x}E_{t}\left(x_{t}\right)$, is a measure of force, so we define it as $F_{t}\left(x_{t}\right)$. Thus, output of a score-based diffusion model can be understood to be a scaled dimensionless force describing a learned energy function, $E_{t}\left(x_{t}\right)$, at time t (see dimensions in Table I):

(13) $$s_{\theta }\left(x_{t},t\right)\approx \nabla_{x}\log p\left(x_{t},t\right)=-\beta \nabla_{x}E_{t}\left(x_{t}\right)=\beta F_{t}\left(x_{t}\right).$$

For samples generated by DFMDock, the data distribution $p_{0}$ aims to capture the distribution of all protein-protein docking systems at equilibrium. $x_{0}$ denotes a docked complex and $Z_{0}=\int_{\Omega }e^{-\beta E\left(x_{0}\right)}dx$ would then be the partition function of two associating proteins. For rigid docking between a receptor protein and a ligand protein, the search space Ω spans all possible translations and rotations of the ligand, with the receptor fixed. Thus, the negative log-likelihood of a docked sample generated at t=0 is, in principle, proportional to the thermodynamic free energy of a protein-protein complex.

### C. Negative Log-Likelihood or “Learned Energy” Estimation

As score-based diffusion models do not produce $p_{0}$ directly, we must find some way to recover it from the neural network’s output: the spatial gradient of the marginal probability distribution, $\nabla_{x}log\;p\left(x_{t},t\right)$. Following [11], we postulate that to recover $p_{0}\left(x_{0}\right)$, one may integrate the change in marginal likelihood along a path from $x_{1}$ in the known prior distribution $p_{1}$ to the sample $x_{0}$ in the unknown data distribution $p_{0}$. We formulate this integral in two ways: first, we use the physical intuition of force and energy to try to construct a work integral over a path through data space x. Second, we use the probability theory behind the marginal distribution of our SDE to formulate an integral of $\frac{\partial log\;p\left(x_{t},t\right)}{\partial t}$ along a path from noise to data. We find that both approaches reduce to solving the same problem: integrating the spatiotemporal gradient field in both space and time of the marginal probability $p\left(x_{t},t\right)$ over a path.

### D. Physics-based Energy Recovery: The Work Integral

In classical mechanics, calculation of the energy accumulated by an object acted on by an external force $F_{t}\left(x_{t}\right)$ over a path S can be achieved using the work integral:

(14) $$\Delta E=-\int_{S}F_{t}\left(x_{t}\right)\cdot dx_{t}.$$

From eq. (13), $F_{t}\left(x_{t},t\right)$ can be replaced by $\beta^{-1}s_{\theta }\left(x_{t},t\right)$ to measure the change in learned energy:

(15) $$\Delta E\approx -\beta^{-1}\int_{S}s_{\theta }\left(x_{t},t\right)\cdot dx_{t}.$$

However, this integral is flawed, and leads to incorrect results both theoretically and numerically. To see why, we simply need to return to the derivation of the work integral:

(16) $$\begin{array}{ll} \Delta E & =\int_{S}\nabla_{x}E\left(x_{t}\right)\cdot dx_{t}\;\text{by}\;\text{F.T.C.}\;\text{for}\;\text{line}\;\text{integrals;}\\ & =\int_{S}-F\left(x_{t}\right)\cdot dx_{t}\;\text{by}\;\text{the}\;\text{definition}\;\text{of}\;\text{force.} \end{array}$$

This derivation makes a fundamental assumption that the energy function, E, is solely a function of space, x, and therefore independent of time, t. However, the connection between learned probability and learned energy gives no such guarantee, as the marginal probability $p\left(x_{t},t\right)$ changes significantly with time. Thus, to compute ∆E, we need to incorporate both the spatial *and* temporal derivatives of E:

(17) $$\begin{array}{ll} dE & =\nabla_{x}E_{t}(x)\cdot dx_{t}+\frac{\partial E_{t}(x)}{\partial t}dt\\ & =-F_{t}\left(x\right)\cdot dx_{t}+\frac{\partial E_{t}\left(x\right)}{\partial t}dt. \end{array}$$

Along a given path $x_{t}$, we can change variables by replacing $dx_{t}$ with $\frac{\partial x_{t}}{\partial t}dt$ to formulate ΔE for a space- and time-dependent $E_{t}\left(x_{t}\right)$:

(18) $$\begin{array}{ll} \Delta E & =\int_{S}dE\\ & =\int_{0}^{1}\left(-F_{t}\left(x_{t}\right)\cdot \frac{\partial x_{t}}{\partial t}+\frac{\partial E_{t}(x)}{\partial t}\right)dt, \end{array}$$

where we now define the integral range from 0 to 1 to correspond to the connection between the data distribution and the noise distribution.

This integral relies on both the spatial and temporal gradients of $E_{t}(x)$. While the learned spatial gradient is easily accessible from the score, how to determine the learned temporal gradient is not obvious. To understand this further, we need to examine the probability theory behind the marginal distribution $p\left(x_{t},t\right)$—so let’s briefly reframe the problem of learned energy recovery to that of likelihood computation.

### E. Negative Log-Likelihood Recovery

Our primary goal is to capture the energy of docked complexes $E_{0}\left(x_{0}\right)$. Since we can directly relate the energy at t=0 with the marginal log-likelihood $log\;p_{0}\left(x_{0}\right)$ via eq. (11), if we can compute this log-likelihood directly, we can subsequently recover the energy up to a constant offset. Replacing E in eq. (18) with logp (and canceling the resulting $\beta^{-1}$ terms) we can consider the line integral of the gradient of this log-likelihood over some path $x_{t}$ through time and space:

(19) $$\Delta \log p=\int_{0}^{1}\left(\nabla_{x}\log p\left(x_{t},t\right)\cdot \frac{\partial x}{\partial t}+\frac{\partial \log p\left(x_{t},\;t\right)}{\partial t}\right)\;dt.$$

The learned score $s_{\theta }\left(x_{t},t\right)$ can be used to approximate $\nabla_{x}log\;p\left(x_{t},t\right)$. The time-derivative $\frac{\partial log\;p}{\partial t}$ can be addressed using the Fokker-Planck equation (see Supplementary Material B), which gives us the change in the marginal probability distribution of an SDE in terms of the diffusion function g and spatial derivatives of the distribution at time t (assuming the drift $\left(x_{t},t\right)=0$):

(20) $$\begin{array}{ll} \frac{\partial log\;p\left(x_{t},t\right)}{\partial t} & =\frac{1}{2}g(t{)}^{2}\left(\nabla_{x}\cdot \nabla_{x}log\;p\left(x_{t},t\right)\right)\\ & +\;\frac{1}{2}g(t{)}^{2}{\left\|\nabla_{x}\log p\left(x_{t},t\right)\right\|}^{2}. \end{array}$$

Now that we can write eq. (19) solely in terms of $\nabla_{x}log\;p\left(x_{t},t\right)$, we can once again substitute the learned score function $s_{\theta }$ from a trained diffusion model. Computing the divergence of the learned score requires taking its derivative; helpfully, modern neural network libraries (in the case of DFMDock, PyTorch) have built-in automatic differentiation capabilities used for backpropagation, so computing this derivative is tractable. Specifically, the divergence of the score is the sum of its partial derivatives along each dimension i, which can be rewritten more compactly as a trace:

$$\begin{array}{ll} \nabla_{x}s_{\theta }\left(x_{t},t\right) & =\sum_{i}\frac{\partial {\left[s_{\theta }\left(x_{t},\;t\right)\right]}_{i}}{\partial {\left[x_{t}\right]}_{i}}\\ & =Tr\left(\nabla_{x}s_{\theta }\left(x_{t},t\right)\right), \end{array}$$

where $\nabla_{x}s_{\theta }\left(x_{t},t\right)$ is the Jacobian matrix of $s_{\theta }$, whose diagonal elements are precisely the derivatives in the sum. Using this identity, we can rewrite eq. (20) in terms of $s_{\theta }$:

(21) $$\begin{array}{ll} \frac{\partial log\;p\left(x_{t},\;t\right)}{\partial t} & \approx \frac{1}{2}g(t{)}^{2}Tr\left(\nabla_{x}s_{\theta }\left(x_{t},t\right)\right)\\ & +\;\frac{1}{2}g(t{)}^{2}{\left\|s_{\theta }\left(x_{t},t\right)\right\|}^{2}. \end{array}$$

Plugging this approximation back into eq. (19), we have an expansion for the change in log probability over some path S in terms of the neural network score. So,

(22) $$\begin{array}{ll} \Delta \;log\;p & \approx \int_{0}^{1}\left(s_{\theta }\left(x_{t},t\right)\cdot \frac{\partial x_{t}}{\partial t}\right.\\ & +\;\frac{1}{2}g(t{)}^{2}Tr\left(\nabla_{x}s_{\theta }\left(x_{t},t\right)\right)\\ & \left.+\;\frac{1}{2}g(t{)}^{2}{\left\|s_{\theta }\left(x_{t},t\right)\right\|}^{2}\right)dt. \end{array}$$

Returning to the energy formulation, eq. (11) tells us the energy field $E_{t}\left(x_{t}\right)$ at time t is proportional to the log marginal probability, $log\;p\left(x_{t},t\right)$, plus the time-independent constant Z, meaning its time derivative is simply

(23) $$\begin{array}{ll} \frac{\partial E_{t}\left(x_{t}\right)}{\partial t} & =-\beta^{-1}\frac{\partial log\;p\left(x_{t},\;t\right)}{\partial t}\\ & \approx -\beta^{-1}\left(\frac{1}{2}g(t{)}^{2}Tr\left(\nabla_{x}s_{\theta }\left(x_{t},t\right)\right)\right.\\ & \left.+\;\frac{1}{2}g(t{)}^{2}{\left\|s_{\theta }\left(x_{t},t\right)\right\|}^{2}\right). \end{array}$$

This completes the energy integral as well:

(24) $$\begin{array}{ll} \Delta E & \approx -\beta^{-1}\int_{0}^{1}\left(s_{\theta }\left(x_{t},t\right)\cdot \frac{\partial x}{\partial t}\right.\\ & +\;\frac{1}{2}g(t{)}^{2}Tr\left(\nabla_{x}s_{\theta }\left(x_{t},t\right)\right)\\ & \left.+\frac{1}{2}g(t{)}^{2}{\left\|s_{\theta }\left(x_{t},t\right)\right\|}^{2}\right)dt\\ & \approx =-\beta^{-1}\Delta \log p. \end{array}$$

In other words: using either the physical intuition or the underlying probability produces a line integral which evaluates the change in learned energy (log likelihood) over some path $x_{t}$. Thus, if we want to compute the learned energy (likelihood) of a point $x_{0}$ in the (unknown) data distribution, it is sufficient to construct a path starting at $x_{0}$ at t=0 and ending at some point $x_{1}$ at $t_{1}$ in the (known) fully noised distribution. Then, using our integral, we can find the log likelihood of $x_{0}$ as

(25) $$\begin{array}{ll} log\;p_{0}\left(x_{0}\right) & \;=log\;p_{1}\left(x_{1}\right)\\ & -\int_{0}^{1}\left(s_{\theta }\left(x_{t},t\right)\cdot \frac{dx_{t}}{dt}\right.\\ & +\;\frac{1}{2}g(t{)}^{2}Tr\left(\nabla_{x}s_{\theta }\left(x_{t},t\right)\right)\\ & \left.+\;\frac{1}{2}g(t{)}^{2}{\left\|s_{\theta }\left(x_{t},t\right)\right\|}^{2}\right)dt. \end{array}$$

The negative sign on the integral stems from the fact that we are integrating from t=0 (data) to t=1 (noise), while the relevant delta is from noise to data. Later, we will be integrating over an ODE trajectory, where the data endpoint is known but the noised endpoint is not, so we ensure consistency by always integrating from t=0 to t=1.

### F. DiffLikelihood: Extracting Log-Likelihood by Integrating Over Sampled Diffusion Paths

The most natural choice of path over which to evaluate our integral is the reverse diffusion trajectory used to generate a given sample, as calculated during model inference. These trajectories are computed by evaluating the reverse SDE equation (4) and using the diffusion model’s output $s_{\theta }\left(x_{t},t\right)$ to approximate $\nabla_{x}log\;p\left(x_{t},t\right)$, resulting in a discrete trajectory of points in data-space $x_{t_{i}}$ and time $t_{i}$. For numerical accuracy while only sampling points on this discrete trajectory, we use the trapezoid rule of numerical integration:

(26) $$\int_{a}^{b}f\left(x\right)dx\approx \left(b-a\right)\frac{f\left(b\right)\;-\;f\left(a\right)}{2}.$$

Applying this to each time step from ($x_{t_{i}},t_{i}$) to $\left(x_{t_{i+1}},t_{i+1}\right)$ produces a discrete equation for $log\;p_{0}\left(x_{0}\right)$

$$\begin{array}{l} log\;p_{0}\left(x_{0}\right)\approx log\;p_{1}\left(x_{1}\right)\\ \;-\sum_{i=1}^{n-1}\frac{1}{2}\left(s_{\theta }\left(x_{t_{i}},t_{i}\right)+s_{\theta }\left(x_{t_{i+1}},t_{i+1}\right)\right)\cdot \Delta x_{t_{i}}\\ \;+\;\frac{1}{4}\left(g{\left(t_{i}\right)}^{2}Tr\left(\nabla_{x}s_{\theta }\left(x_{t_{i}},t_{i}\right)\right)\right.\\ \;+\;g{\left(t_{i}\right)}^{2}{\left\|s_{\theta }\left(x_{t_{i}},t_{i}\right)\right\|}^{2}\\ \;+\;g{\left(t_{i+1}\right)}^{2}Tr\left(\nabla_{x}s_{\theta }\left(x_{t_{i+1}},t_{i+1}\right)\right)\\ \;\left.\;+g{\left(t_{i+1}\right)}^{2}{\left\|s_{\theta }\left(x_{t_{i+1}},t_{i+1}\right)\right\|}^{2}\right)\Delta t_{i}, \end{array}$$

where $\Delta x_{t_{i}}=x_{t_{i+1}}-x_{t_{i}}$ and $\Delta t_{i}=t_{i+1}-t_{i}$.

The reverse diffusion path is convenient because it allows likelihood integration concurrent with inference by reusing the score computed by the network. Likewise, the trapezoid rule is the highest order numerical method that doesn’t require sampling additional intermediate points (and thus additional evaluations of the score model). By using the trapezoid rule once per pair of vertices, we effectively approximate the integral of a piecewise-differentiable path of line segments. If need be, one could integrate each segment more accurately by further interpolating to intelligently sample the score at more points along the trajectory. We find this extra precision does not significantly change the results for either of our test cases, implying that using the trapezoid rule achieves sufficient numerical accuracy along each segment (data not shown).

**FIG. 2.**
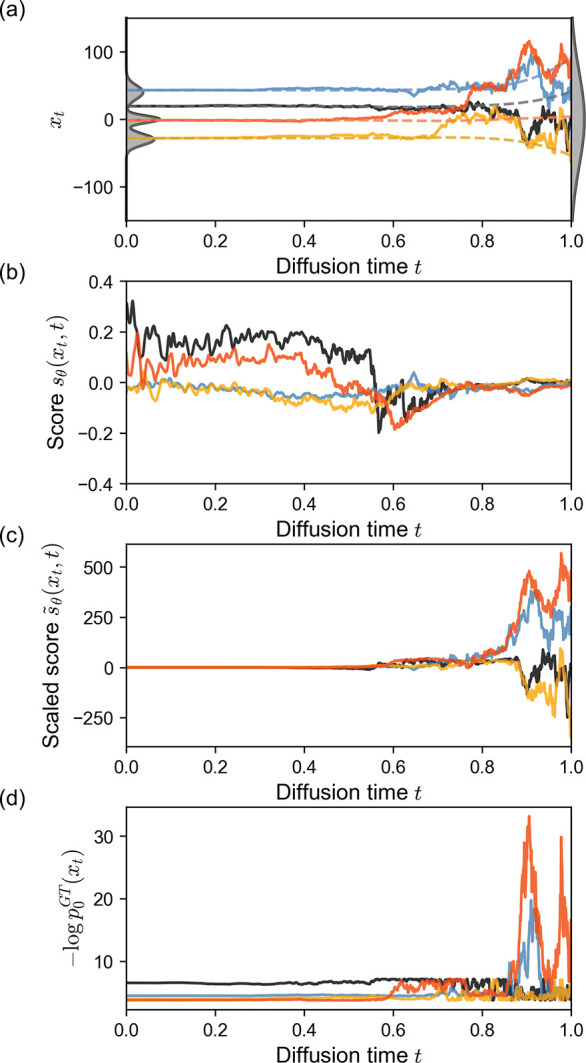
Trajectories for four individual 1D samples. (a) Diffusion trajectories $x_{t}$ from the Gaussian mixture diffusion model. Flow paths that result in the same ending points as the diffusion paths are shown as dashed lines. Ground-truth distribution (left) and prior distribution (right) shown in gray. (b) The learned score function $s_{\theta }\left(x_{t},t\right)=\nabla_{x}log\;p\left(x_{t},t\right)$. (c) The scaled score function ${\tilde{s}}_{\theta }\left(x_{t},t\right)=-\frac{1}{2}g(t{)}^{2}\nabla_{x}log\;p\left(x_{t},t\right)$ with the exponential noise schedule $g(t)=\sigma_{0}{\left(\frac{\sigma_{1}}{\sigma_{0}}\right)}^{t}$. (d) Ground truth energy $-log\;p_{0}^{GT}\left(x_{t}\right)$ evaluated over 1000 diffusion steps from t=1 to t=0.

**FIG. 3.**
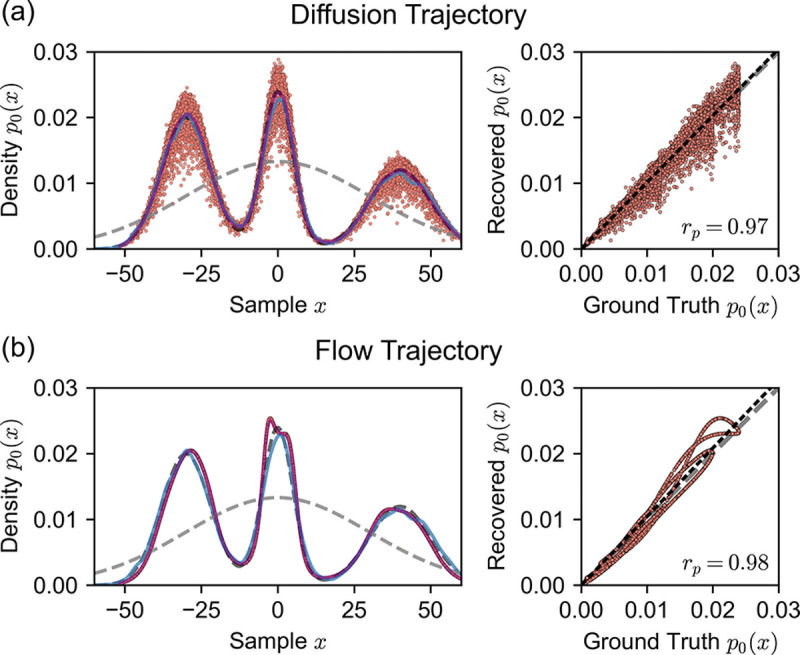
Recovered probabilities $p_{0}\left(x_{0}\right)=e^{-\beta E_{0}\left(x_{0}\right)}$ for samples generated from a 1D Gaussian mixture diffusion model integrated over (a) diffusion trajectories and (b) flow trajectories (red dots, left panels). For the diffusion trajectories in (a), the binned mean of recovered probabilities is shown as a purple curve. The dashed black curve represents the data distribution $p_{0}\left(x_{0}\right)$, the dashed gray curve shows the unimodal Gaussian distribution (the prior) $p_{1}\left(x_{1}\right)$, and the blue curve is a kernel density estimate of samples (N=10,000) generated from the diffusion model. The right panels show correlation plots of recovered probability values from both trajectories versus the ground truth probability values. A y=x line is shown in gray dashes, and a dotted linear model is shown in black with Pearson coefficients $r_{p}$.

Despite this, we will see the noise term in eq. (4) produces paths with large displacements $\Delta x_{t}$ (Fig. 2a) and noisy likelihoods (Fig. 3a), motivating us to explore integration over other paths.

### G. FlowLikelihood: Extracting Log-Likelihood by Integrating over Flow-Equivalent ODEs

Since $log\;p\left(x_{t},t\right)$ is a state function, we are free to integrate over arbitrary paths with the same endpoints. A less noisy alternative to the diffusion trajectories is to use a flow-equivalent ODE as described by [11], where paths are generated from the following ODE:

(27) $$\frac{dx_{t}}{dt}=-\frac{1}{2}g(t{)}^{2}\nabla_{x}log\;p\left(x_{t},t\right)\approx -\frac{1}{2}g(t{)}^{2}s_{\theta }\left(x_{t},t\right).$$

The likelihood integral in eq. (25) is then evaluated over these flow trajectories from t=0 to t=1 with initial condition $x_{0}$, the point in the data distribution whose learned likelihood we seek.

The explicit description of the path in terms of an ODE confers two major benefits over a discretely sampled diffusion trajectory: First, both path and integrand can be computed using existing black-box ODE solvers, rather than the limited trapezoidal method used over the diffusion trajectory. Second, since we have an explicit form for $dx_{t}$, we can substitute it directly into our integrand’s $dx_{t}$ term. When we do so, the first and third terms cancel (see Supplementary Material C), leaving us with the much simplified:

(28) $$\begin{array}{ll} log\;p_{0}\left(x_{0}\right) & =log\;p_{1}\left(x_{1}\right)-\int_{0}^{1}\frac{1}{2}g(t{)}^{2}\left(\nabla_{x}\cdot \nabla_{x}log\;p\left(x_{t},t\right)\right)dt\\ & \approx log\;p_{1}\left(x_{1}\right)-\int_{0}^{1}\frac{1}{2}g(t{)}^{2}Tr\left(\nabla_{x}s_{\theta }\left(x_{t},t\right)\right)dt. \end{array}$$

Interestingly, this result also follows from interpreting the process of 1) sampling from the prior and 2) following the probability-flow ODE as a continuous normalizing flow model as introduced by Chen *et al.* in [38]; Song *et al.* [11] used this derivation path when first introducing likelihood calculation for diffusion models.

Integrating over the flow trajectory simplifies the integral and provides a smoother, easier to integrate path than the noisy, discrete diffusion trajectory. However, it comes at the computational cost of having to generate the path for each sample. Unlike diffusion trajectory integration, since a flow trajectory can be computed for any point in the data space, we are no longer limited to assessing the likelihood of points generated during inference.

Pseudocode algorithms for integration over diffusion (Supp. Algorithm 1) and flow (Supp. Algorithm 2) trajectories can be found in Supplementary Material D.

## IV. RESULTS

### A. Temporal Analysis of Diffusion Trajectories from 1D Diffusion Model

To validate our theoretical analysis, we trained a 1D diffusion model to learn a simple Gaussian mixture model (see Section VI for training procedure), generated samples from our learned model, and evaluated the recovery of learned energy over both reverse diffusion and flow trajectories.

Fig. 2a shows four sampled reverse diffusion trajectories. The diffusion trajectories from the model are noisy at early times (t=1) and become progressively smoother as the model gradually transforms the points to the data distribution (t→0) and the amount of added noise per diffusion step decreases. By solving the probability flow ODE (eq. 27), we also compute and show the flow paths that result in the same sample point at t=0 (dashed lines). Fig. 2b shows the learned score function $s_{\theta }\left(x_{t},t\right)=\nabla_{x}log\;p\left(x_{t},t\right)$ along those four diffusion trajectories. The score has low magnitude near t=1 which indicates low confidence of steering towards the high-likelihood regions of the data. As time decreases, the magnitude of the score increases, and the model shows stronger guidance for $x_{t}$ towards high-likelihood regions of the data distribution.

In Fig. 2c, we visualize the scaled score-term used in the reverse SDE, ${\tilde{s}}_{\theta }\left(x_{t},t\right)=-\frac{1}{2}g(t{)}^{2}\nabla_{x}log\;p\left(x_{t},t\right)$, where g(t) is the noise-schedule (diffusion coefficient). As we use an exponential noise schedule $g(t)=\sigma_{0}{\left(\frac{\sigma_{1}}{\sigma_{0}}\right)}^{t}$ which exhibits strong weighting, ${\tilde{s}}_{\theta }\left(x_{t},t\right)$ shows large fluctuations near t=1, and its magnitude decays as t→0. To better interpret how the system evolves from noise to data, we use the ground truth distribution of the trimodal Gaussian to plot $-log\;p_{0}^{GT}\left(x_{t}\right)$ evaluated over the 1000 reverse diffusion steps (Fig. 2d). The ground truth energies are high and noisy at early times, but the fluctuations gradually decay and converge to a stable value as $x_{t}$ approaches the high-likelihood regions of the data distribution $p_{0}\left(x_{0}\right)$.

### B. Integration Over Diffusion and Flow Trajectories Recover Probabilities that Approximate the True Data Distribution

In Fig. 3a-b, we plot the 1D system ground truth (training) distribution $p_{0}\left(x_{0}\right)$ (dashed black curve) and the prior distribution $p_{1}\left(x_{1}\right)$ (dashed gray curve). To check the 1D diffusion model’s ability to generate samples from the data distribution, we plot the kernel density estimate (KDE) of 10,000 sample data points generated from the model (blue curve), showing that the model has learned the ground truth distribution well (Kolmogorov-Smirnov distance of 0.012).

Next, we examined the energies recovered from our integral formulations. We computed negative log-likelihood values (NLL) of samples generated by our Gaussian mixture diffusion model by integrating over both diffusion and flow trajectories. To evaluate the quality of the NLL values, we plot the recovered probabilities $p_{0}\left(x_{0}\right)=e^{-E_{0}\left(x_{0}\right)}$ (red dots in Fig. 3a-b). The distribution recovered by integrating over diffusion trajectories shows noisy deviation from the training data distribution, but the binned average probability (purple line) matches the ground-truth training distribution almost exactly. In contrast, integrating over flow trajectories yields a noise-free smooth curve that closely follows the ground-truth distribution. Flow-integrated likelihoods demonstrate consistent skew error near the central peak, something not seen when integrating over diffusion trajectories.

To quantify the consistency between the integrals, Fig. 3 shows scatter plots of the recovered probability versus ground truth probability for the three integral methods (right panels). The Pearson correlation coefficients ($r_{p}=0.970$ for diffusion and $r_{p}=0.979$ for flow) indicate that learned likelihoods from both diffusion and flow trajectories approximate the underlying data distribution well.

### C. Temporal Analysis of Protein Docking Diffusion Trajectories from DFMDock

Following the 1D diffusion model analysis, we now apply the same approaches to a 3D, rigid-body, translational diffusion model for protein docking, where the diffusion process occurs over $x_{t}=(x,y,z)$ in 3D Euclidean space. We performed our analysis on the DFMDock model [20], which learns to dock protein complexes given two unbound protein monomers. We limited the docking space to translation only (see Section VI), as the Fokker-Planck equation used assumes $R^{n}$ Euclidean space and is incompatible with general Riemannian manifolds like the SE(3) space used in rotational diffusion. We performed DFMDock inference on 25 targets from Docking Benchmark 5.5 (DB5.5) [43, 44] with 120 samples for each target.

**FIG. 4.**
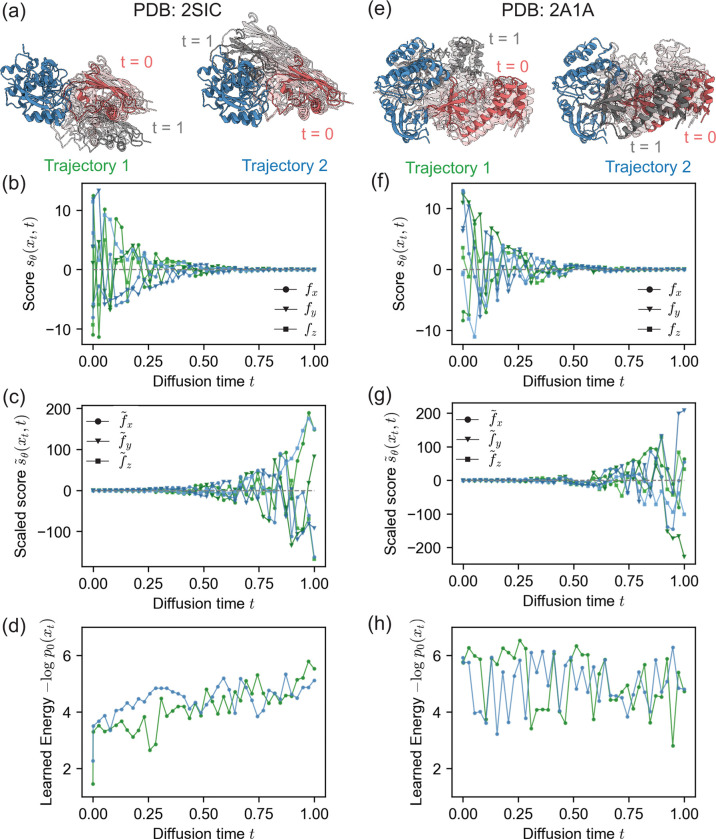
DFMDock reverse diffusion trajectories for two successful docking samples of two protein complexes, (a-d) 2SIC and (e-h) 2A1A. (a,e) Top two DockQ structures. Receptor protein, blue; ligand protein, gray (t=1) to red (t=0). (b,f) Score function learned by DFMDock $s_{\theta }\left(x_{t},t\right)=\nabla_{x}log\;p\left(x_{t},t\right)$ along x,y, and z directions. (c,g) Scaled score function ${\tilde{s}}_{\theta }\left(x_{t},t\right)=-\frac{1}{2}g(t{)}^{2}\nabla_{x}log\;p\left(x_{t},t\right)$ with $g(t)=\sigma_{0}{\left(\frac{\sigma_{1}}{\sigma_{0}}\right)}^{t}$ along x, y, and z directions. (d,h) Learned energy, $-log\;p_{0}\left(x_{t}\right)$, recovered from integrating flow trajectories terminating at each point along the reverse diffusion trajectory.

We first examined the score and the learned energies over the 40-step reverse diffusion trajectories starting from the unbound monomers at t=1 to the bound complex at t=0 for two example targets. As an example of a complex for which DFMDock generated accurate samples, we selected the complex of subtilisin BPN’ with an inhibitor (PDB: 2SIC). DFMDock-generated samples of docked 2SIC structures exhibited DockQ scores of up to 0.96 (high quality). As an example of a complex for which DFMDock generated diverse but low-accuracy structures, we chose PKR kinase domain-eIF2*α* (PDB: 2A1A); the best generated docked 2A1A structure only reached a DockQ score of 0.71, a medium quality ranking. Two denoising trajectories from t=1 to t=0 representing the most successful docking attempts for each target are shown in Fig. 4a,e. Similar to 1D cases, we observe increasing score $s_{\theta }\left(x_{t},t\right)$ (Fig. 4b,f) and decreasing scaled score ${\tilde{s}}_{\theta }\left(x_{t},t\right)$ (Fig. 4c,g) moving towards t=0.

As flow trajectories can be used to retrieve the energy of a pose without needing its diffusion trajectory, we used them to compute the learned energy, $-log\;p_{0}\left(x_{t}\right)$, of intermediate poses $x_{t}$ under the learned data distribution $p_{0}$ (Fig. 4d,f). For the 2SIC trajectories, the learned energy shows a general downward trend over the course of 40 diffusion steps, suggesting that the reverse diffusion process increasingly favored more energetically plausible protein-protein interactions learned by the model. This decrease in energy can be correlated with improved docking quality as the model samples more refined conformations. The 2A1A trajectories, on the other hand, display deep and sharp oscillations and few general trends as the diffusion time approaches zero, notably *not* decreasing as time goes to zero.

### D. Learned Energies Compare to Rosetta Energy for Scoring Protein-Protein Interactions

We then computed the learned energy $-log\;p_{0}\left(x_{0}\right)$ of 120 DFMDock generated structures for all 25 protein-protein complexes. As in the 1D case, we extracted potentials for each sample by integrating eqs. (25) and (28) over diffusion and flow trajectories respectively (Supp. Fig. S1-S4). To analyze whether these potentials are low for near-native structures, we used two measures of similarity to the ground truth structures, interface-residue RMSD (Supp. Fig. S1, S3) and DockQ (a composite measure for docking quality, Supp. Fig. S2, S4).

**FIG. 5.**
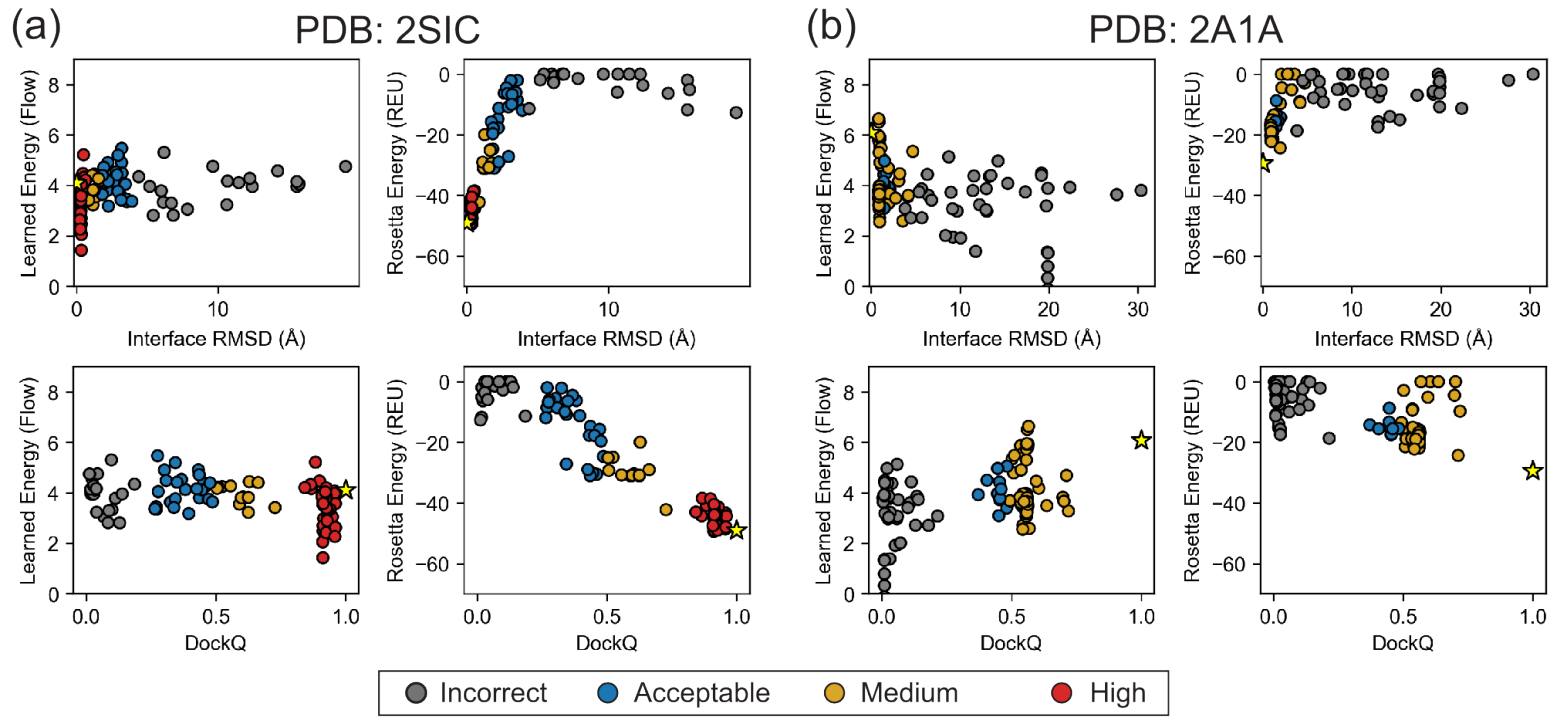
Learned energy (recovered from integrating over flow trajectories) and Rosetta energy of 120 generated docking poses plotted against interface RMSD (top) or DockQ (bottom) for (a) PDB ID: 2SIC and (b) PDB ID: 2A1A. Individual points are colored by the quality of the corresponding docking pose based on the CAPRI classification [45]: incorrect, gray; acceptable, blue; medium quality, gold; high quality, red. Energies computed for the ground truth structures are shown as yellow stars.

Given that the 1D case showed the learned energy recovered from diffusion trajectories were noisy, we focus on the learned docking energy recovered from flow trajectories. A comparison of the recovery of DFMDock learned energies over flow and diffusion trajectories can be found in Supp. Fig. S9. Additionally, we calculated the interface energies using Rosetta, a leading physics-inspired biomolecular modeling package, for all 25 targets (Supp. Figs. S5, S6). Here, we focus on our analysis of the learned energies for the same two example targets analyzed in Section IV.C, 2SIC and 2A1A (Fig. 5).

For both 2SIC and 2A1A, the Rosetta energy reveals a funnel-like curve with the global minimum near the ground truth (native) structure. The learned energy for 2SIC shares a near-native global minimum (Fig. 5a). DFMDock’s 2SIC energy landscape seems to have a sharp funnel centered near the native structure, with a relatively flat distribution outside of it. That is, many samples lie at approximately the same non-minimal energy ($\beta E_{0}\approx 4$), including several samples ranked well by DockQ or interface RMSD. The inability to distinguish between medium- and low-quality structures might indicate that DFMDock has learned little about physical long-range interactions that might guide a protein towards its docked conformation, instead learning that structures without proper short-range interactions are unlikely to be native.

In the case of 2A1A, the DFMDock learned energies fail to capture the correct binding energetics, with the minimum-energy samples largely being incorrect (iRMSD ≈ 20, DockQ ≈ 0), while Rosetta energy correctly ranks near-native poses best. The contrast between DFMDock’s ability to sample high-quality poses and its inability to properly rank poses with its energy for certain targets indicates that the DFMDock learned energy does not reflect the physical principles of protein-protein interactions in all cases. When the learned energy of the ground truth poses (Fig. 5, Supp. Fig. S3, S4, yellow stars) are compared to those of the generated poses, they are often higher than the best docking pose and fall within two standard deviations of lower quality ones. The high learned energies of ground truth poses suggests that DFMDock has not sufficiently learned to capture protein-protein interactions.

**FIG. 6.**
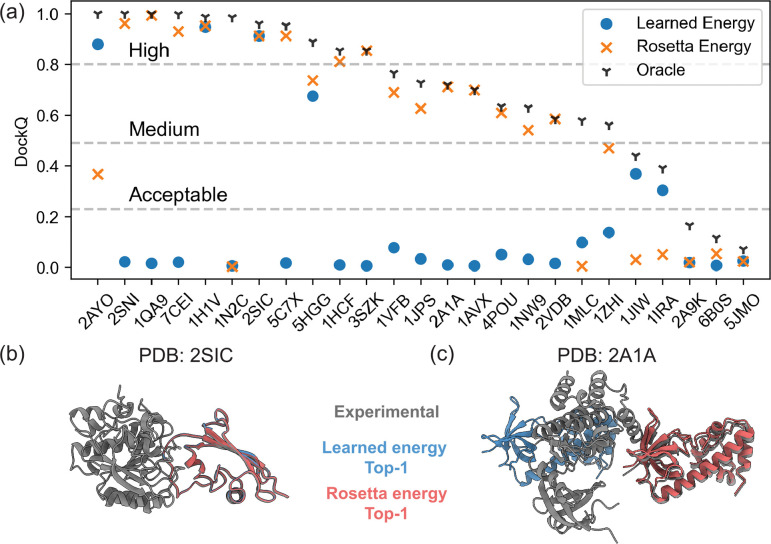
(a) Comparison of top-ranked model quality (DockQ) for 25 targets from the DB5.5 test set with learned energy over flow trajectories (blue circles), Rosetta energy (orange crosses), or in the oracle setting (black triangle). Top predictions ranked by the learned energy (blue) and Rosetta energy (red) for (b) 2SIC and (c) 2A1A.

We next explored whether the funnel-like behavior and the orthogonality of the DFMDock learned energy is useful for ranking docking poses. Fig. 6a compares the top ranked model quality (DockQ) for the 25 protein-protein complexes with the learned energy, Rosetta energy, and an oracle setting (where we rank poses based on their DockQ score relative to the ground truth pose). The learned energy performs comparably or outperforms Rosetta interface energy in 6 out of 25 cases in identifying correct docking poses among the sampled poses. Fig. 6b,c shows a structural comparison between the learned energy and Rosetta interface energy’s top-ranked predictions for two targets. For 2SIC, both the learned energy and Rosetta interface energy identify (distinct) high quality poses, with DockQ = 0.91 in each case. However, for 2A1A, the learned energy fails to identify even an acceptable quality structure (DockQ = 0.01) while Rosetta energy identifies a medium quality pose (DockQ = 0.71).

## V. DISCUSSION AND CONCLUSION

The goal of this work was to interpret diffusion models through the lens of statistical thermodynamics by analyzing the underlying learned potential function (at t=0) and exploring its applications in scoring protein complexes. We developed theory rooted in statistical thermodynamics to relate the probability of observing a system in a particular state to the energy of that state, the inverse temperature β. We defined the energy function that a diffusion model implicitly learns as the *learned energy* and showed that it is equal to the negative log-likelihood, $-log\;p_{0}\left(x_{0}\right)$, up to a constant reference energy logZ.

Since diffusion models do not explicitly learn the data distribution $p_{0}\left(x_{0}\right)$, but rather the score function, $\nabla_{x}log\;p\left(x_{t},t\right)$, we developed methods to evaluate $p_{0}\left(x_{0}\right)$. We postulated that to recover $p_{0}\left(x_{0}\right)$, we can integrate along a path from the known prior distribution $p_{1}\left(x_{1}\right)$ to the target data distribution, $p_{0}\left(x_{0}\right)$. We constructed the path integral over $x_{t}$ to calculate the likelihood, and evaluated it over two paths: (1) discretized diffusion trajectories from the reverse SDE, and (2) flow trajectories, which follow a smooth, deterministic path defined by an ODE equivalent in marginal probability to the reverse SDE.

We initially tested our approach on a simple 1D diffusion model of a Gaussian mixture and evaluated $-log\;p_{0}\left(x_{0}\right)$ and $p_{0}\left(x_{0}\right)$ values of samples generated by the model using our integral methods and compared them to the known analytical solution. While diffusion trajectory integrals are convenient since they can be calculated from model inference points, their integration results in noisy likelihood recovery, even as smoothed likelihoods well approximate the ground truth distribution. Fortunately, flow trajectories are both smooth and integrable using black-box ODE solvers. Flow trajectories effectively and continuously approximate the probabilities of the true data distribution at the cost of an additional path calculation.

Building on these results, we applied the same methodology to a protein-protein diffusion docking model, DFMDock, which generates protein complex structures given two unbound protein monomer structures. We extracted the learned potentials from DFMDock to score protein complexes and compared them to Rosetta energies. The learned energies from DFMDock-generated docking poses reveal binding energy funnels that sometimes match Rosetta’s interface energy funnels in that the near-native structures have lower energies. But in other cases, the learned energy funnels have minima at non-native structures, and the native structures have higher energies. This work shows that we can examine ensembles of diffusion-generated protein structures in a similar manner as in physics-based energy approaches.

An open question in the field of protein structure prediction and design is whether and how AI models might learn a thermodynamic function of protein folding and association. Better interpretability of AI models in biology is also important from a biosecurity perspective. There are other investigations of these questions in the literature. Ahdritz *et al.* [46] released OpenFold, an open-source version of AF2 and carefully investigated model’s learning process and the model’s capacity to generalize to unseen regions of fold space. Roney *et al.* [47] hypothesized that AF2 has learned an implicit energy function through its confidence module, and demonstrated that AF2 can be used to rank the quality of candidate protein structures without needing coevolution data.

To our knowledge, this work is the first attempt at extracting potentials from a biomolecular diffusion model and comparing them with physical energy functions. An exciting future direction is to apply our methodology developed here on state-of-the-art diffusion-based structure prediction models such as AlphaFold3 [7] or Boltz-2 [14] to interrogate their learned energy functions associated with protein folding stability. Another important direction is to generalize likelihood calculation to be compatible with diffusion models over arbitrary Riemannian manifolds such as the SE(3) space used in the original DFMDock with rotations [20] and frame-based models like DiffDock-PP [26]. As we are not limited only to computing the likelihood of generated samples, exploring the learned energy landscape around generated structures might reveal important details about the shape of the learned energy funnel. For example, whether the structure is at a local minimum could be used as a ranking metric, and learned energy surfaces could be used for local refinement.

While we explored here how physical energies can be accessed from learned diffusion models, another approach is to inject biophysical knowledge into AI models. Kulyte *et al.* [48] used molecular dynamics (MD) force-fields to guide diffusion models for antibody design. Similarly, Wang *et al.* [49] used MD force-fields to guide diffusion models for protein conformation generation. Lewis *et al.* [50] trained a diffusion model on MD conformational ensembles to improve realistic diversity of conformational sampling. Analyses like ours could help probe the effects of adding physical information into deep-learning based biomolecular design and structure prediction tools.

Our method for likelihood calculation integrates the spatiotemporal gradient of the marginal probability $p\left(x_{t},t\right)$ over two paths: the probability flow ODE, and a discrete sampling of the reverse-time SDE. Interestingly, this gradient is (by definition) a conservative vector field over the combined diffusion space $R^{n+1}$ (combining data-space $x_{t}\in R^{n}$ and time t∈R), which means that the integral of this gradient, ∆logp, should be dependent solely on the endpoints of the path, not on the trajectory we use for integration. Since we have an explicit formula for $log\;p_{1}$, this means the calculated value of $log\;p_{0}=log\;p_{1}-\Delta log\;p$ should be the same for any path we use during integration.

In practice, however, we find this is not the case: flow trajectories do not yield the same value as diffusion trajectories, and different diffusion paths terminating at near-identical samples can have significantly different likelihoods. There is evidence that diffusion models do not generally learn spatially-conservative [51] or Fokker-Planck consistent [52] scores, and so the accuracy of the score may vary significantly depending on the similarity of a sample to data seen during training. There may be further insight to be gained by exploring the conservativity in the score learned by networks like DFMDock and AlphaFold and the impacts inaccuracies have on different trajectories and methods of likelihood computation.

## VI. DATA

### A. Gaussian Mixture Model Training

To train a 1D diffusion model to learn a simple Gaussian mixture distribution, we generated 60,000 training data points from a trimodal Gaussian, defined as a weighted sum of three individual Gaussian components. The probability density function is

(29) $$\begin{array}{ll} P_{0}(x)= & w_{1}\cdot 𝒩\left(x;\mu_{1},b_{1}^{2}\right)+w_{2}\cdot 𝒩\left(x;\mu_{2},b_{2}^{2}\right)\\ & \;+\;w_{3}\cdot 𝒩\left(x;\mu_{3},b_{3}^{2}\right), \end{array}$$

where the means are $\mu_{1}=-30$, $\mu_{2}=0$, and $\mu_{3}=40$; the standard deviations are $b_{1}=8.0$, $b_{2}=5.0$, and $b_{3}=10.0$; and the mixture weights are $w_{1}=0.4$, $w_{2}=0.3$, and $w_{3}=0.3$. For testing, we sampled 10,000 synthetic points with 1,000 diffusion steps from the learned model and compared the probability density function of the analytical formulation with the kernel density estimate of the generated samples (Fig. 3).

### B. DFMDock Inference

We performed inference using the DFMDock model [20] which is trained on DIPS-hetero [53], a subset of DIPS with approximately 11,000 heterodimers. Although DFMDock was originally designed to reverse both translational and rotational noise, here we restricted the added rigid-body noise during both training and inference to be translation-only, such that all processes occur in the 3D Euclidean space $R^{3}$. Additionally, the original formulation of DFMDock uses a random sampling method to construct the interaction graph as input to the neural network, whereas here the graph is generated deterministically using k-nearest neighbors. This ensures the stochasticity during inference comes solely from added perturbations. Using the same training parameters as [20], we retrained the model with these modifications and observed slightly degraded performance. As in [20], we sampled 120 poses with 40 diffusion steps on 25 targets from the Docking Benchmark 5.5 (DB5.5) [43, 44], a widely used dataset for assessing docking performance. For Rosetta energies computed for the sampled poses, we first performed the local_docking_refine protocol before computing the $I_{sc}$ score with the REF15 energy function.

## Supplementary Material

Supplement 1

## Figures and Tables

**TABLE I.** Mathematical terms of diffusion models and their interpretations as AI or physics jargon. Example units: m, meters; J, Joules. λ is used as an arbitrary unit of diffusion “time.” Dimensions: T, physical time; τ, diffusion/alchemical time; L, length; M, mass. Dimensional analysis deriving these units from the major equations is provided in Section A of the Supplemental Material.

Term	AI interpretation	Physical transformation	Unit	Dimension

t	Diffusion time	Alchemical transformation variable	λ	[τ]
$x_{t}$	State	Position	m	[L]
β=1/kT	Scaling constant	Inverse temperature	$J^{-1}$	$[T{]}^{2}[M{]}^{-1}{\left[L\right]}^{-2}$
$E_{t}\left(x_{t}\right)$	Time-dependent energy	Energy	J	$[M][L{]}^{2}{\left[T\right]}^{-2}$
$\nabla_{x}\;log\;p\left(x_{t},t\right)$	Score	-	$m^{-1}$	$[L{]}^{-1}$
$\beta^{-1}\nabla_{x}\;log\;p\left(x_{t},t\right)$	-	Force	$Jm^{-1}$	$[M][L]{\left[T\right]}^{-2}$
$g{\left(t\right)}^{2}$	Diffusion coefficient	-	$m^{2}\lambda^{-1}$	$[L{]}^{2}{\left[\tau \right]}^{-1}$
g(t)	Scheduler	-	$m\lambda^{-1/2}$	$[L]{\left[\tau \right]}^{-1/2}$
$w_{t}∼𝒩(0,I)$	Perturbation kernel	Thermal fluctuations	$\lambda^{1/2}$	${\left[T\right]}^{1/2}$
$f\left(x_{t},t\right)$	Drift	External potential	$m\lambda^{-1}$	$[L]{\left[\tau \right]}^{-1}$
$-log\;p_{0}\left(x_{0}\right)$	Negative log-likelihood	-	1	[1]
$-\beta^{-1}\;log\;p_{0}\left(x_{0}\right)$	-	Thermodynamic free energy	J	$[M][L{]}^{2}{\left[T\right]}^{-2}$

## CODE AVAILABILITY

The training, inference, and likelihood evaluation code for the 1D diffusion model and the likelihood evaluation code for DFMDock are available at https://github.com/Graylab/DiffEnergy.
