# Supplementary material for: Can We Extract Physics-like Energies from Generative Protein Diffusion Models?
Source: bioRxiv. 2025 Dec 17:2025.11.28.690021. Originally published 2025 Nov 29. Preprint. [Version 3] doi: 10.1101/2025.11.28.690021 (PMC12697549; doi:10.1101/2025.11.28.690021)
Supplement: Supplement 1 [file NIHPP2025.11.28.690021v3-supplement-1.pdf]

bioRxiv preprint doi: https://doi.org/10.1101/2025.11.28.690021; this version posted December 17, 2025. The copyright holder for this preprint (which was not certified by peer review) is the author/funder, who has granted bioRxiv a license to display the preprint in perpetuity. It is made available under aCC-BY 4.0 International license.

SUPPORTING INFORMATION

A. Dimensional Analysis of Key Equations

We perform dimensional analysis on the fundamental equations underlying diffusion (and their physics counterparts) to derive the units for **Table I**; $[M]$ represents units of mass, $[L]$ units of length, $[T]$ units of *physical* time, and $[\tau]$ units of *diffusion/alchemical* time. We begin with the forward diffusion equation, eq. (3):

$$dx_t = f(x_t, t)dt + g(t)dw_t.$$

where x_t represents a point in Euclidean space $\mathbb{R}^n$ and thus has units of length $[L]$. More generally, x_t can be a point on an arbitrary Riemannian manifold; every such manifold has a Riemannian metric, a measure of distance between two points, so units of length still broadly apply. The Wiener term dw_t is sampled from a Gaussian with variance dt, defined in terms of alchemical/diffusion time, meaning its units must be $[\tau]^{1/2}$, the square root of the unit of the variance. The units of the f and g functions can thus be inferred:

$$dx_t \sim [L], \qquad dt \sim [\tau], \qquad dw_t \sim [\tau]^{1/2},$$

$$f(x_t, t) \sim \frac{[L]}{[\tau]}, \qquad \text{“Drift”}$$

$$g(t) \sim \frac{[L]}{[\tau]^{1/2}}. \qquad \text{“Scheduler”}$$

The reverse diffusion equation, eq. (4),

$$dx_t = \left[f(x_t, t) - g(t)^2 \nabla_x \log p(x_t, t)\right] dt + g(t)dw_t$$

introduces the marginal probability $p(x_t, t)$, which is unitless, and reveals the units of $g(t)^2$ and $\nabla_x \log p(x_t, t)$:

$$p(x_t, t) \sim [1],$$

$$g(t)^2 \sim \frac{[L]^2}{[\tau]}, \qquad \text{“Diffusion Coefficient”}$$

$$\nabla_x \log p(x_t, t) \sim \frac{1}{[L]}. \qquad \text{“Score”}$$

Equations (10) and (11) connect the marginal probability to energy:

$$p(x_t, t) = \frac{e^{-\beta E_t(x_t)}}{Z};$$
$$\log p(x_t, t) = -\beta E_t(x_t) - \log Z.$$

These equations tell us $\beta E_t(x_t)$ and Z must be unitless, and the dimensions of energy are defined by physics:

$$Z \sim [1], \quad E_t(x_t) \sim \frac{[M][L]^2}{[T]^2}, \quad \beta \sim \frac{[T]^2}{[M][L]^2},$$

$$-\beta^{-1} \log p(x_t, t) \sim \frac{[M][L]^2}{[T]^2}. \qquad \text{“Free Energy”}$$

Finally, equation (13) connects energy and physics:

$$s_\theta(x_t, t) \approx \nabla_x \log p(x_t, t) = -\beta \nabla_x E_t(x_t),$$

introducing learned score $s_\theta(x_t, t)$ and force $-\nabla_x E_t(x_t)$:

$$s_\theta(x_t, t) \sim \frac{1}{[L]}, \qquad \text{“Learned Score”}$$

$$\nabla_x E_t(x_t) = -\beta^{-1} \nabla_x \log p(x_t, t) \sim \frac{[M][L]}{[T]^2}. \qquad \text{“Force”}$$

For completeness, we also include dimensional analysis of our integral formulations. For eq. (19):

$$\Delta \log p = \int_0^1 \left(\nabla_x \log p(x_t, t) \cdot \frac{\partial x_t}{\partial t} + \frac{\partial \log p(x_t, t)}{\partial t} \right) dt,$$

$$\frac{\partial x_t}{\partial t} \sim \frac{[L]}{[\tau]}, \qquad \frac{\partial \log p(x_t, t)}{\partial t} \sim \frac{[1]}{[\tau]}.$$

And eq. (20):

$$\frac{\partial \log p(x_t, t)}{\partial t} = \frac{1}{2} g(t)^2 \Big(\nabla_x \cdot \nabla_x \log p(x_t, t) \Big) + \frac{1}{2} g(t)^2 \|\nabla_x \log p(x_t, t)\|^2,$$

$$\frac{1}{2} g(t)^2 \Big(\nabla_x \cdot \nabla_x \log p(x_t, t) \Big) \sim \frac{[L]^2}{[\tau]} \cdot \frac{[1]}{[L]^2} = \frac{[1]}{[\tau]},$$

$$\frac{1}{2} g(t)^2 \|\nabla_x \log p(x_t, t)\|^2 \sim \frac{[L]^2}{[\tau]} \cdot \frac{[1]}{[L]^2} = \frac{[1]}{[\tau]}.$$

bioRxiv preprint doi: https://doi.org/10.1101/2025.11.28.690021; this version posted December 17, 2025. The copyright holder for this preprint (which was not certified by peer review) is the author/funder, who has granted bioRxiv a license to display the preprint in perpetuity. It is made available under aCC-BY 4.0 International license.

B. Derivation of Logarithmic Fokker Planck Equation

The Fokker-Planck equation is a partial differential equation (PDE) that describes the time-evolution of the marginal probability distribution $p(x_t, t)$ associated with a given stochastic differential equation (SDE). It has many variants depending on the form of the SDE; for $dx_t = f(x_t, t)dt + g(t)dw_t$ the Fokker-Planck equation is [54]

$$\begin{aligned}\frac{\partial p(x_t,t)}{\partial t} &= -\sum_{i=1}^{n} \frac{\partial}{\partial [x_t]_i}\Big([f(x_t,t)]_i\ p(x_t,t)\Big) \\ &+ \frac{1}{2}g(t)^2 \sum_{i=1}^{n} \frac{\partial^2}{\partial [x_t]_i^2} p(x_t,t),\end{aligned} \tag{S-1}$$

where n is the dimensionality of x_t, $[x_t]_i$ is the i-th component of x_t, and $[f(x_t,t)]_i$ is the i-th component of the drift term $f(x_t,t)$.

The Fokker-Planck equation is useful, but it is dependent on the value and spatial gradients of probability $p(x_t,t)$, neither of which we have; rather, our score model is trained to predict the spatial gradients of the log probability. We can convert between the two using the chain rule:

$$\begin{aligned}\frac{\partial \log f(x)}{\partial x_i} &= \frac{d\log f(x)}{df(x)}\frac{\partial f(x)}{\partial x_i} \\ &= \frac{1}{|f(x)|}\frac{\partial f(x)}{\partial x_i}.\end{aligned}$$

Since $p(x_t,t) > 0$,

$$\frac{\partial \log p(x_t,t)}{\partial t} = \frac{1}{p(x_t,t)}\frac{\partial p(x_t,t)}{\partial t}. \tag{S-2}$$

Thus, we can rewrite the Fokker-Planck equation (S-1) in a logarithmic form like so:

$$\begin{aligned}\frac{\partial \log p(x_t,t)}{\partial t} &= \frac{1}{p(x_t,t)}\frac{\partial p(x_t,t)}{\partial t} \\ &= -\frac{1}{p(x_t,t)}\sum_{i=1}^{n} \frac{\partial}{\partial [x_t]_i}\Big([f(x_t,t)]_i\ p(x_t,t)\Big) \\ &+ \frac{1}{p(x_t,t)}\frac{1}{2}g(t)^2 \sum_{i=1}^{n} \frac{\partial^2}{\partial [x_t]_i^2} p(x_t,t).\end{aligned} \tag{S-3}$$

This equation is still dependent on derivatives of $p(x_t,t)$, but we can convert them into logarithmic derivatives as well by multiplying by $\frac{1}{p(x_t,t)}$. The first-order term is relatively straightforward; first, we expand the derivative using the product rule:

$$\begin{aligned}\frac{\partial \log p(x_t,t)}{\partial t} &= -\frac{1}{p(x_t,t)}\sum_{i=1}^{n} p(x_t,t)\left(\frac{\partial}{\partial [x_t]_i}[f(x_t,t)]_i\right) \\ &- \frac{1}{p(x_t,t)}\sum_{i=1}^{n} [f(x_t,t)]_i\left(\frac{\partial}{\partial [x_t]_i}p(x_t,t)\right) \\ &+ \frac{1}{p(x_t,t)}\frac{1}{2}g(t)^2 \sum_{i=1}^{n} \frac{\partial^2}{\partial [x_t]_i^2}\left(p(x_t,t)\right).\end{aligned} \tag{S-4}$$

In the first term, $p(x_t,t)$ cancels, and in the second term, the $p(x_t,t)$ derivative becomes logarithmic by eq. (S-2):

$$\begin{aligned}\frac{\partial \log p(x_t,t)}{\partial t} &= -\sum_{i=1}^{n} \frac{\partial}{\partial [x_t]_i}[f(x_t,t)]_i \\ &- \sum_{i=1}^{n} [f(x_t,t)]_i\left(\frac{\partial}{\partial [x_t]_i}\log p(x_t,t)\right) \\ &+ \frac{1}{p(x_t,t)}\frac{1}{2}g(t)^2 \sum_{i=1}^{n} \frac{\partial^2}{\partial [x_t]_i^2} p(x_t,t).\end{aligned} \tag{S-5}$$

bioRxiv preprint doi: https://doi.org/10.1101/2025.11.28.690021; this version posted December 17, 2025. The copyright holder for this preprint (which was not certified by peer review) is the author/funder, who has granted bioRxiv a license to display the preprint in perpetuity. It is made available under aCC-BY 4.0 International license.

The second-order derivative in the third term requires more work. We can again use eq. (S-2) to derive an identity for logarithmic second derivatives:

$$\begin{aligned}
\frac{\partial^2}{\partial [x_t]_i^2} p(x_t,t) &= \frac{\partial}{\partial [x_t]_i}\left(\frac{\partial}{\partial [x_t]_i} p(x_t,t)\right) \\
&= \frac{\partial}{\partial [x_t]_i}\left(\left(\frac{\partial}{\partial [x_t]_i}\log p(x_t,t)\right)\ p(x_t,t)\right) \\
&= \left(\frac{\partial^2}{\partial [x_t]_i^2}\log p(x_t,t)\right) p(x_t,t) + \left(\frac{\partial}{\partial [x_t]_i}\log p(x_t,t)\right)\left(\frac{\partial}{\partial [x_t]_i} p(x_t,t)\right) \\
&= \left(\frac{\partial^2}{\partial [x_t]_i^2}\log p(x_t,t)\right) p(x_t,t) + \left(\frac{\partial}{\partial [x_t]_i}\log p(x_t,t)\right)\left(\frac{\partial}{\partial [x_t]_i}\log p(x_t,t)\right) p(x_t,t) \\
&= \left(\frac{\partial^2}{\partial [x_t]_i^2}\log p(x_t,t)\right) p(x_t,t) + \left(\frac{\partial}{\partial [x_t]_i}\log p(x_t,t)\right)^2 p(x_t,t).
\end{aligned} \tag{S-6}$$

Substituting this back into equation (S-5), the problematic term splits into a second-order logarithmic term and a squared first-order term, and the final remaining $p(x_t,t)$ coefficients cancel:

$$\begin{aligned}
\frac{\partial \log p(x_t,t)}{\partial t} &= -\sum_{i=1}^{n}\frac{\partial}{\partial [x_t]_i}[f(x_t,t)]_i \\
&\quad - \sum_{i=1}^{n}[f(x_t,t)]_i\left(\frac{\partial}{\partial [x_t]_i}\log p(x_t,t)\right) \\
&\quad + \frac{1}{2}g(t)^2\sum_{i=1}^{n}\frac{\partial^2}{\partial [x_t]_i^2}\log p(x_t,t) \\
&\quad + \frac{1}{2}g(t)^2\sum_{i=1}^{n}\left(\frac{\partial}{\partial [x_t]_i}\log p(x_t,t)\right)^2,
\end{aligned} \tag{S-7}$$

giving us a form of the logarithmic Fokker-Planck equation that we can evaluate using only a trained score model. These sums can also be written using vector notation:

$$\begin{aligned}
\frac{\partial \log p(x_t,t)}{\partial t} &= -\nabla_x \cdot f(x_t,t) \\
&\quad - f(x_t,t)\cdot\nabla_x \log p(x_t,t) \\
&\quad + \frac{1}{2}g(t)^2\nabla_x\cdot\nabla_x \log p(x_t,t) \\
&\quad + \frac{1}{2}g(t)^2\|\nabla_x \log p(x_t,t)\|^2.
\end{aligned} \tag{S-8}$$

Finally, since our models are variance-exploding, their drift term $f(x_t,t)=0$, simplifying to eq. (20) in the main text:

$$\begin{aligned}
\frac{\partial \log p(x_t,t)}{\partial t} &= \frac{1}{2}g(t)^2\Big(\nabla_x\cdot\nabla_x \log p(x_t,t)\Big) \\
&\quad + \frac{1}{2}g(t)^2\|\nabla_x \log p(x_t,t)\|^2.
\end{aligned} \tag{20}$$

B.1 Reverse-Time Fokker-Planck

Technically, the process whose marginal distribution we seek is the *reverse* diffusion process, eq. (4):

$$dx_t = [f(x_t,t) - g(t)^2\nabla_x \log p(x_t,t)]dt + g(t)d\overline{w}_t. \tag{4}$$

As mentioned earlier, the reverse diffusion process is carefully chosen such that its marginal distribution matches that of the forward process, and therefore the Fokker-Planck equations should be identical for both (in fact, Anderson (1982) [41] derived the reverse SDE precisely by matching the Fokker-Planck for the forward and backward equations). For thoroughness, we will also derive the marginal distribution for the reverse process.

bioRxiv preprint doi: https://doi.org/10.1101/2025.11.28.690021; this version posted December 17, 2025. The copyright holder for this preprint (which was not certified by peer review) is the author/funder, who has granted bioRxiv a license to display the preprint in perpetuity. It is made available under aCC-BY 4.0 International license.

The most important thing to keep track of is the direction of time and the sign of its change. The Fokker-Planck equation assumes a positive dt and a forward-time Wiener process dw_t, but the reverse SDE involves a negative dt and a reverse-time Wiener process $d\overline{w}_t$. To adjust, we perform change-of-variables on t to produce an equivalent forward-time process in the variable s; by replacing t in eq. (4) with $1-s$, changing the sign of the first term to account for replacing $dt < 0$ with $ds > 0$, and using the forward-time Wiener term:

$$dx_{1-s} = [-f(x_{1-s}, 1-s) + g(1-s)^2 \nabla_x \log p(x_{1-s}, 1-s)]ds + g(1-s)dw_s. \tag{S-9}$$

The logarithmic Fokker-Planck equation for this SDE then describes the change in the reverse marginal probability, $\overline{p}$, with respect to s:

$$\begin{aligned}
\frac{\partial \log \overline{p}(x_{1-s}, 1-s)}{\partial s} &= -\nabla_x \cdot [-f(x_{1-s}, 1-s) + g(1-s)^2 \nabla_x \log p(x_{1-s}, 1-s)] \\
&- [-f(x_{1-s}, 1-s) + g(1-s)^2 \nabla_x \log p(x_{1-s}, 1-s)] \cdot \nabla_x \log \overline{p}(x_{1-s}, 1-s) \\
&+ \frac{1}{2} g(1-s)^2 \nabla_x \cdot \nabla_x \log \overline{p}(x_{1-s}, 1-s) \\
&+ \frac{1}{2} g(1-s)^2 \|\nabla_x \log \overline{p}(x_{1-s}, 1-s)\|^2 \\
\\
&= \nabla_x \cdot f(x_{1-s}, 1-s) - g(1-s)^2 \nabla_x \cdot \nabla_x \log p(x_{1-s}, 1-s) \\
&+ f(x_{1-s}, 1-s) \cdot \nabla_x \log \overline{p}(x_{1-s}, 1-s) - g(1-s)^2 \nabla_x \log p(x_{1-s}, 1-s) \cdot \nabla_x \log \overline{p}(x_{1-s}, 1-s) \\
&+ \frac{1}{2} g(1-s)^2 \nabla_x \cdot \nabla_x \log \overline{p}(x_{1-s}, 1-s) \\
&+ \frac{1}{2} g(1-s)^2 \|\nabla_x \log \overline{p}(x_{1-s}, 1-s)\|^2 \\
\\
&= \nabla_x \cdot f(x_{1-s}, 1-s) \\
&+ f(x_{1-s}, 1-s) \cdot \nabla_x \log \overline{p}(x_{1-s}, 1-s) \\
&+ g(1-s)^2 \nabla_x \cdot \nabla_x \left(\frac{1}{2} \log p(x_{1-s}, 1-s) - \log \overline{p}(x_{1-s}, 1-s) \right) \\
&+ g(1-s)^2 \nabla_x \log \overline{p}(x_{1-s}, 1-s) \cdot \nabla_x \left(\frac{1}{2} \log p(x_{1-s}, 1-s) - \log \overline{p}(x_{1-s}, 1-s) \right).
\end{aligned} \tag{S-10}$$

To compare with the Fokker-Planck for the forward process, eq. (S-8), we'd like the derivative with respect to t, not s. Re-substituting $1-s$ with t and using the chain rule:

$$\begin{aligned}
\frac{\partial \log \overline{p}(x_{1-s}, 1-s)}{\partial s} &= \frac{\partial \log \overline{p}(x_t, t)}{\partial (1-t)} \\
&= -\frac{\partial \log \overline{p}(x_t, t)}{\partial t},
\end{aligned} \tag{S-11}$$

which means the temporal gradient of the reverse process in terms of t is:

$$\begin{aligned}
\frac{\partial \log \overline{p}(x_t, t)}{\partial t} &= -\frac{\partial \log \overline{p}(x_{1-s}, 1-s)}{\partial s} \\
&= -\nabla_x \cdot f(x_t, t) \\
&- f(x_t, t) \cdot \nabla_x \log \overline{p}(x_t, t) \\
&+ g(t)^2 \nabla_x \cdot \nabla_x \left(\log \overline{p}(x_t, t) - \frac{1}{2} \log p(x_t, t) \right) \\
&+ g(t)^2 \nabla_x \log \overline{p}(x_t, t) \cdot \nabla_x \left(\log \overline{p}(x_t, t) - \frac{1}{2} \log p(x_t, t) \right).
\end{aligned} \tag{S-12}$$

bioRxiv preprint doi: https://doi.org/10.1101/2025.11.28.690021; this version posted December 17, 2025. The copyright holder for this preprint (which was not certified by peer review) is the author/funder, who has granted bioRxiv a license to display the preprint in perpetuity. It is made available under aCC-BY 4.0 International license.

To show that $p(x_t,t) = \overline{p}(x_t,t)$, then, it is sufficient to show that $p(x_t,t)$ is a solution to eq. (S-12) and that both functions satisfy the same initial value problems. That $p(x_t,t)$ satisfies eq. (S-12) is easy to show by substituting $p(x_t,t)$ for $\overline{p}(x_t,t)$ and observing that it simplifies to the forward logarithmic Fokker-Planck equation, eq. (S-8):

$$\begin{aligned}\frac{\partial \log \overline{p}(x_t,t)}{\partial t} &= -\nabla_x \cdot f(x_t,t) \\ &- f(x_t,t)\cdot\nabla_x \log p(x_t,t) \\ &+ g(t)^2\nabla_x\cdot\nabla_x\left(\log p(x_t,t) - \frac{1}{2}\log p(x_t,t)\right) \\ &+ g(t)^2\nabla_x \log p(x_t,t)\cdot\nabla_x\left(\log p(x_t,t) - \frac{1}{2}\log p(x_t,t)\right) \qquad \text{(S-13)} \\ &= -\nabla_x \cdot f(x_t,t) \\ &- f(x_t,t)\cdot\nabla_x \log p(x_t,t) \\ &+ \frac{1}{2}g(t)^2\nabla_x\cdot\nabla_x \log p(x_t,t) \\ &+ \frac{1}{2}g(t)^2\|\nabla_x \log p(x_t,t)\|^2, \qquad \text{(S-8)}\end{aligned}$$

and the initial condition, $\overline{p}(x_1,1) = p_1(x_1)$, is satisfied by our assumption that we add sufficient noise for $p(x_1,1) = p_1(x_1)$. Since $p(x_t,t)$ satisfies the differential equation for $\overline{p}(x_t,t)$ with the same initial conditions, we conclude that $p(x_t,t) = \overline{p}(x_t,t)$ as expected. To save on this algebraic manipulation in the main body of the paper, we always use the Fokker-Planck of the forward equation and assume $dt > 0$.

C. Derivation of Flow Trajectory Integral

Applying eq. (25) to the flow ODE path, we can simplify the equation analytically by subbing in the path's $\frac{dx_t}{dt}$ as defined by the ODE, eq. (27):

$$\begin{aligned}\frac{dx_t}{dt} &= -\frac{1}{2}g(t)^2\nabla_x \log p(x_t,t) \qquad (27) \\ &\approx -\frac{1}{2}g(t)^2 s_\theta(x_t,t).\end{aligned}$$

Substituting this into eq. (25):

$$\log p_0(x_0) = \log p_1(x_1) - \int_0^1 \left(s_\theta(x_t,t)\cdot\frac{dx_t}{dt} + \frac{1}{2}g(t)^2\mathrm{Tr}\Big(\nabla_x s_\theta(x_t,t)\Big) + \frac{1}{2}g(t)^2\|s_\theta(x_t,t)\|^2\right)dt \qquad (25)$$

$$= \log p_1(x_1) - \int_0^1 \left(s_\theta(x_t,t)\cdot\left(-\frac{1}{2}g(t)^2 s_\theta(x_t,t)\right) + \frac{1}{2}g(t)^2\mathrm{Tr}\Big(\nabla_x s_\theta(x_t,t)\Big) + \frac{1}{2}g(t)^2\|s_\theta(x_t,t)\|^2\right)dt$$

$$= \log p_1(x_1) - \int_0^1 \left(-\cancel{\frac{1}{2}g(t)^2\|s_\theta(x_t,t)\|^2} + \frac{1}{2}g(t)^2\mathrm{Tr}\Big(\nabla_x s_\theta(x_t,t)\Big) + \cancel{\frac{1}{2}g(t)^2\|s_\theta(x_t,t)\|^2}\right)dt$$

$$= \log p_1(x_1) - \int_0^1 \frac{1}{2}g(t)^2\mathrm{Tr}\Big(\nabla_x s_\theta(x_t,t)\Big)dt. \qquad (28)$$

bioRxiv preprint doi: https://doi.org/10.1101/2025.11.28.690021; this version posted December 17, 2025. The copyright holder for this preprint (which was not certified by peer review) is the author/funder, who has granted bioRxiv a license to display the preprint in perpetuity. It is made available under aCC-BY 4.0 International license.

D. Pseudocodes for Log Likelihood Integrals

These are pseudocode algorithms for integrating over discrete diffusion trajectories using the trapezoid rule (Algorithm 1) and flow trajectories using black-box ODE solvers (Algorithm 2).

Algorithm 1 Trapezoidal Integration over Diffusion Trajectories

Input: Diffusion trajectory samples $\{(x_{t_0}, t_0), (x_{t_1}, t_1), \ldots, (x_{t_n}, t_n)\}$ where $t_i \in [0,1], t_0 = 0, t_1 = 1$; score model $s_\theta(x_t, t)$; noise schedule $g(t)$
Output: Log-likelihood estimate $\log p_0(x_0) = \log p_1(x_1) - \Delta \log p$
function INTEGRAND(x_t, t,Δx, Δt)
    return $s_\theta(x_t, t) \cdot \Delta x + \frac{1}{2} g(t)^2 \mathrm{Tr}\Big(\nabla_x s_\theta(x_t, t)\Big) + \frac{1}{2} g(t)^2 \|s_\theta(x_t, t)\|^2$
end function
Initialize $\Delta \log p \leftarrow 0$
for $i = 0$ to $n - 1$ **do**
    $\Delta x_i \leftarrow x_{t_{i+1}} - x_{t_i}$
    $\Delta t_i \leftarrow t_{i+1} - t_i$
    $f_a \leftarrow \mathrm{Integrand}(x_{t_i}, t_i, \Delta x_i, \Delta t_i)$
    $f_b \leftarrow \mathrm{Integrand}(x_{t_{i+1}}, t_{i+1}, \Delta x_i, \Delta t_i)$
    $\Delta \log p \leftarrow \Delta \log p + \frac{1}{2}(f_a + f_b)$
end for
Compute $\log p_1(x_1) \sim \mathcal{N}(0, I)$ ▷ Gaussian prior
$\log p_0(x_0) \leftarrow \log p_1(x_1) - \Delta \log p$
return $\log p_0(x_0)$

Algorithm 2 ODE Integration over Flow Paths

Input: Diffusion sample x_0; score model $s_\theta(x_t, t)$; noise schedule $g(t)$
Output: Log-likelihood $\log p_0(x_0)$
function DRIFT(x_t, t)
    return $-g(t)^2 \cdot s_\theta(x_t, t)$
end function
function TRACEJACOBIAN(x_t, t)
    return $\frac{1}{2} g(t)^2 \mathrm{Tr}\Big(\nabla_x s_\theta(x_t, t)\Big)$
end function
Solve the following ODEs from $t = 0$ to $t = 1$ using an ODE solver:

- $\frac{dx_t}{dt} = \mathrm{Drift}(x_t, t)$ with initial condition x_0
- $\frac{d(\log p)}{dt} = \mathrm{TraceJacobian}(x_t, t)$ with initial value 0
- **return** $\{x_t|_{t=0}^{1}\}, \Delta \log p$

Compute $\log p_1(x_1) \sim \mathcal{N}(0, I)$ ▷ Gaussian prior
$\log p_0(x_0) \leftarrow \log p_1(x_1) - \Delta \log p$
return $\log p_0(x_0)$

bioRxiv preprint doi: https://doi.org/10.1101/2025.11.28.690021; this version posted December 17, 2025. The copyright holder for this preprint (which was not certified by peer review) is the author/funder, who has granted bioRxiv a license to display the preprint in perpetuity. It is made available under aCC-BY 4.0 International license.

E. Additional Supplementary Figures

Supp. Fig.	y-axis	x-axis
Supp. Fig. S1	Diffusion Trajectory Learned Energy	Interface RMSD
Supp. Fig. S2	Diffusion Trajectory Learned Energy	DockQ
Supp. Fig. S3	Flow Trajectory Learned Energy	Interface RMSD
Supp. Fig. S4	Flow Trajectory Learned Energy	DockQ
Supp. Fig. S5	Rosetta Energy	Interface RMSD
Supp. Fig. S6	Rosetta Energy	DockQ
Supp. Fig. S7	Flow Trajectory Learned Energy	Rosetta Energy
Supp. Fig. S8	Diffusion Trajectory Learned Energy	Rosetta Energy
Supp. Fig. S9	Diffusion Trajectory Learned Energy	Flow Trajectory Learned Energy

bioRxiv preprint doi: https://doi.org/10.1101/2025.11.28.690021; this version posted December 17, 2025. The copyright holder for this preprint (which was not certified by peer review) is the author/funder, who has granted bioRxiv a license to display the preprint in perpetuity. It is made available under aCC-BY 4.0 International license.

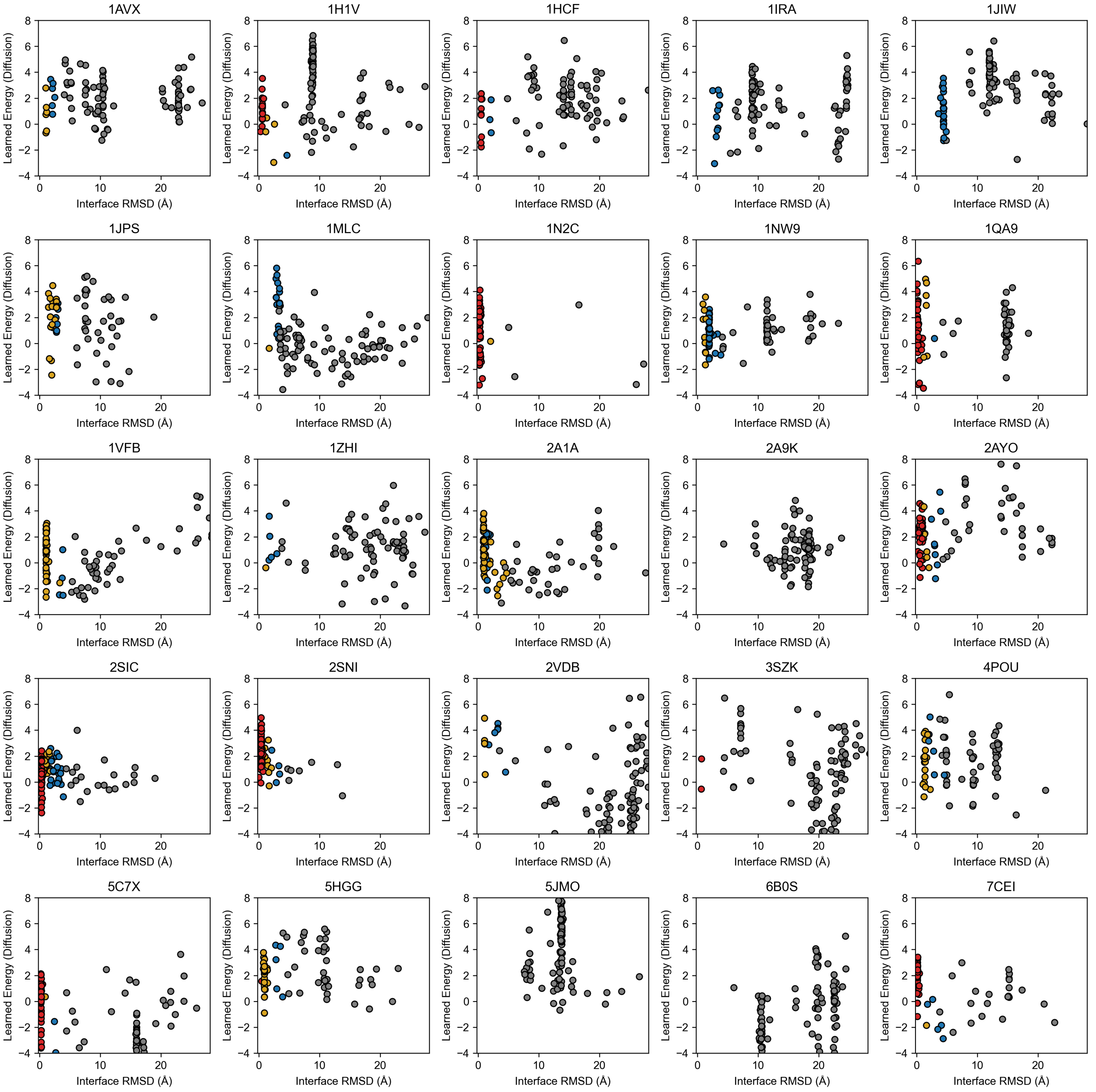

FIG. S1. Learned energy computed from integrating over diffusion trajectories, plotted against interface RMSD of 120 docking poses generated from DFMDock for 25 targets in the DB5.5 dataset. Individual points are colored by their docking quality based on the CAPRI classification (incorrect: gray, acceptable: blue, medium: gold, high: red).

bioRxiv preprint doi: https://doi.org/10.1101/2025.11.28.690021; this version posted December 17, 2025. The copyright holder for this preprint (which was not certified by peer review) is the author/funder, who has granted bioRxiv a license to display the preprint in perpetuity. It is made available under aCC-BY 4.0 International license.

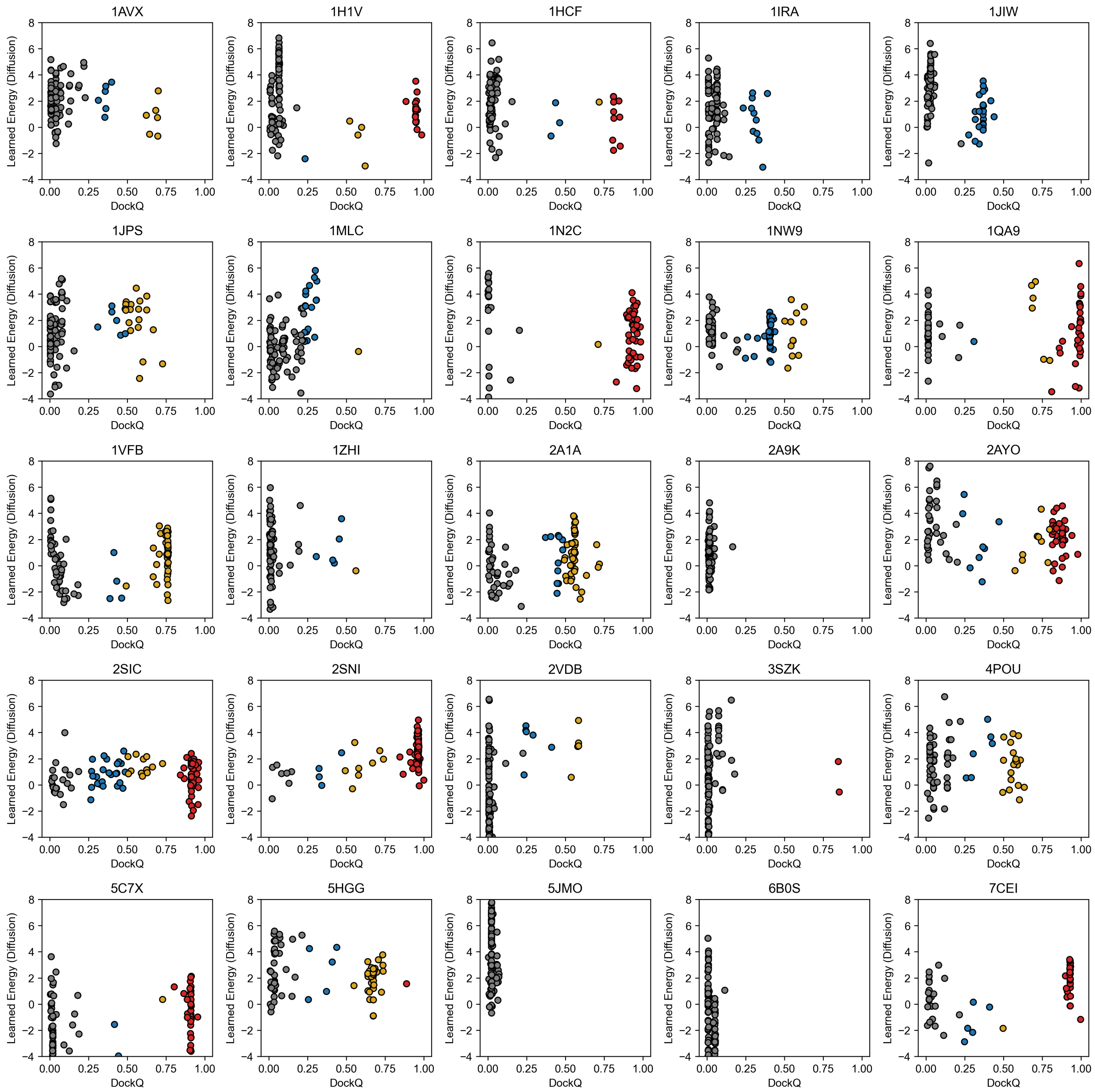

FIG. S2. Learned energy computed from integrating over diffusion trajectories, plotted against DockQ of 120 docking poses generated from DFMDock for 25 targets in the DB5.5 dataset. Individual points are colored by their docking quality based on the CAPRI classification (incorrect: gray, acceptable: blue, medium: gold, high: red).

bioRxiv preprint doi: https://doi.org/10.1101/2025.11.28.690021; this version posted December 17, 2025. The copyright holder for this preprint (which was not certified by peer review) is the author/funder, who has granted bioRxiv a license to display the preprint in perpetuity. It is made available under aCC-BY 4.0 International license.

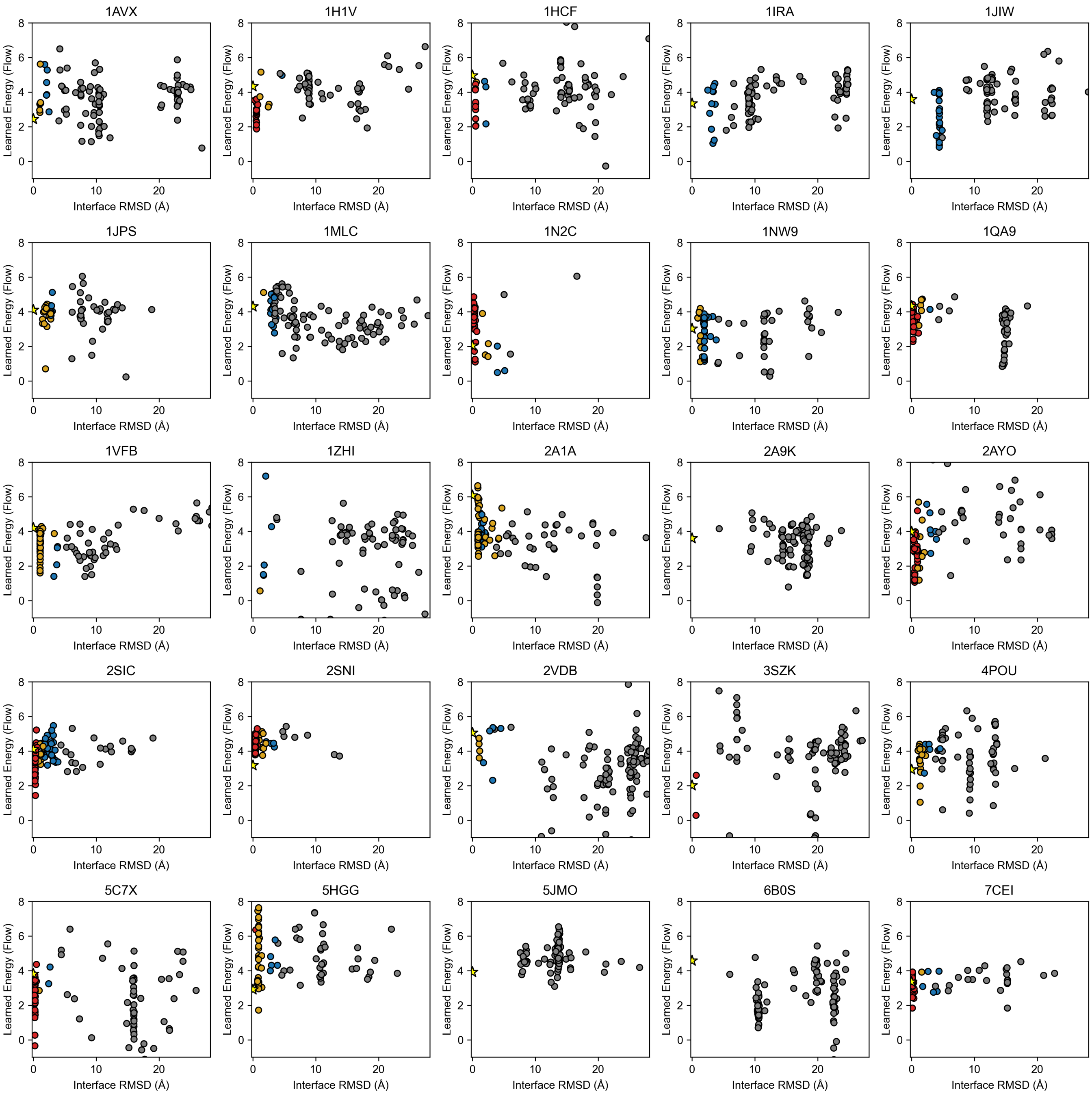

FIG. S3. Learned energy computed from integrating over flow trajectories, plotted against interface RMSD of 120 docking poses generated from DFMDock for 25 targets in the DB5.5 dataset. Individual points are colored by their docking quality based on the CAPRI classification (incorrect: gray, acceptable: blue, medium: gold, high: red). Energy of the ground truth structure is shown as a yellow star.

bioRxiv preprint doi: https://doi.org/10.1101/2025.11.28.690021; this version posted December 17, 2025. The copyright holder for this preprint (which was not certified by peer review) is the author/funder, who has granted bioRxiv a license to display the preprint in perpetuity. It is made available under aCC-BY 4.0 International license.

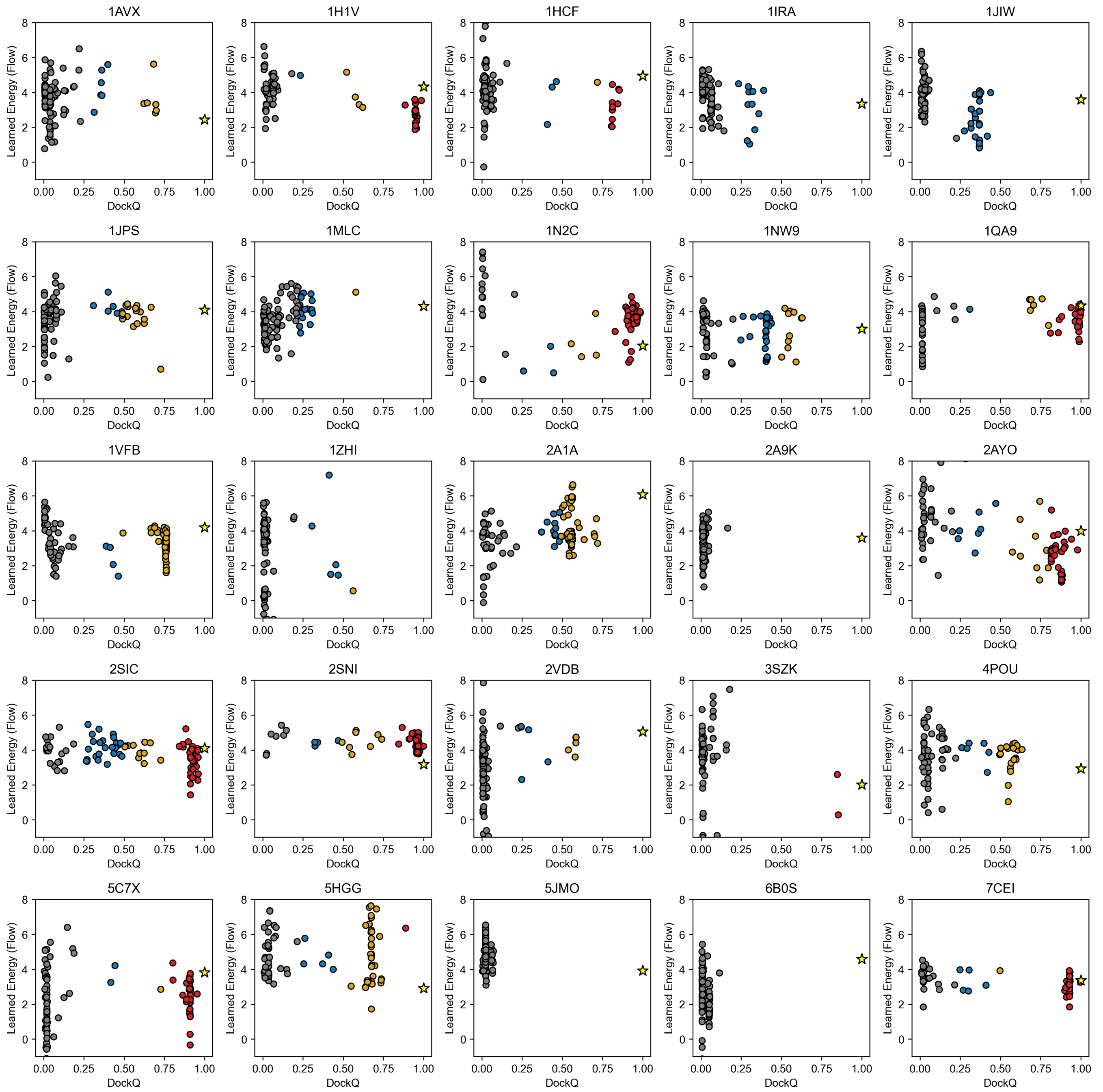

FIG. S4. Learned energy computed from integrating over flow trajectories, plotted against DockQ of 120 docking poses generated from DFMDock for 25 targets in the DB5.5 dataset. Individual points are colored by their docking quality based on the CAPRI classification (incorrect: gray, acceptable: blue, medium: gold, high: red). Energy of the ground truth structure is shown as a yellow star.

bioRxiv preprint doi: https://doi.org/10.1101/2025.11.28.690021; this version posted December 17, 2025. The copyright holder for this preprint (which was not certified by peer review) is the author/funder, who has granted bioRxiv a license to display the preprint in perpetuity. It is made available under aCC-BY 4.0 International license.

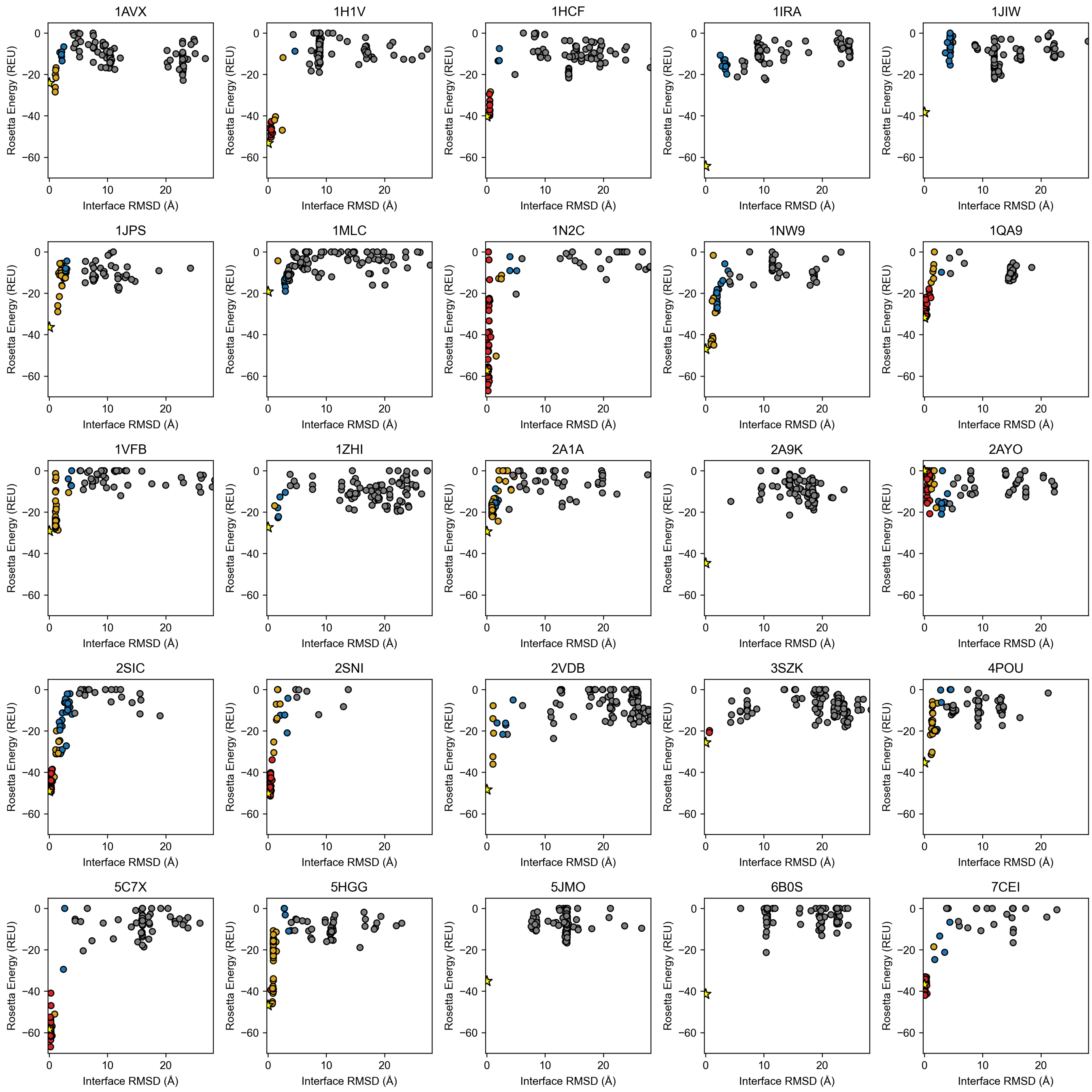

FIG. S5. Rosetta energy plotted against interface RMSD of 120 docking poses generated from DFMDock for 25 targets in the DB5.5 dataset. Individual points are colored by their docking quality based on the CAPRI classification (incorrect: gray, acceptable: blue, medium: gold, high: red). Energy of the ground truth structure is shown as a yellow star.

bioRxiv preprint doi: https://doi.org/10.1101/2025.11.28.690021; this version posted December 17, 2025. The copyright holder for this preprint (which was not certified by peer review) is the author/funder, who has granted bioRxiv a license to display the preprint in perpetuity. It is made available under aCC-BY 4.0 International license.

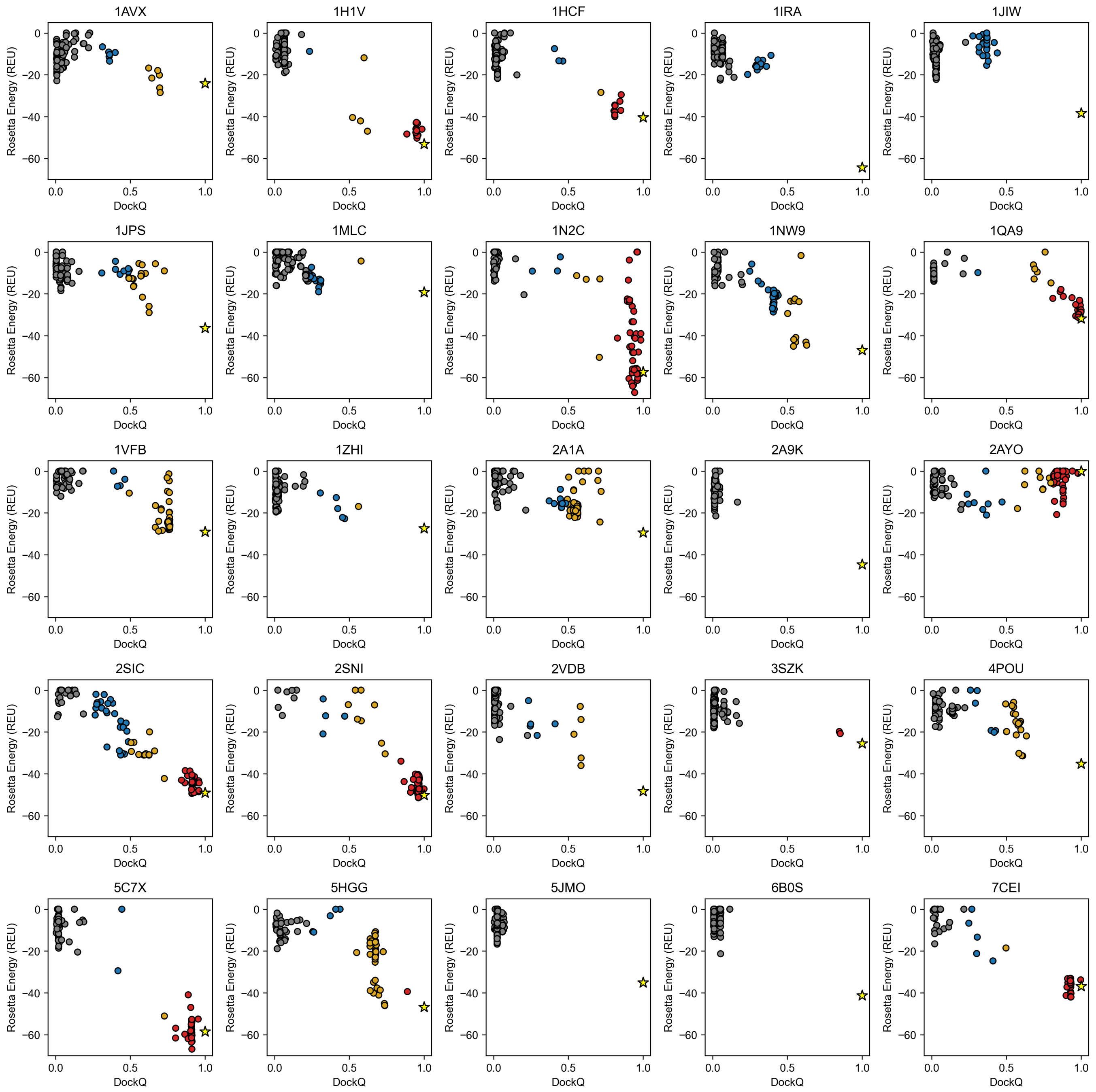

FIG. S6. Rosetta energy plotted against DockQ of 120 docking poses generated from DFMDock for 25 targets in the DB5.5 dataset. Individual points are colored by their docking quality based on the CAPRI classification (incorrect: gray, acceptable: blue, medium: gold, high: red). Energy of the ground truth structure is shown as a yellow star.

bioRxiv preprint doi: https://doi.org/10.1101/2025.11.28.690021; this version posted December 17, 2025. The copyright holder for this preprint (which was not certified by peer review) is the author/funder, who has granted bioRxiv a license to display the preprint in perpetuity. It is made available under aCC-BY 4.0 International license.

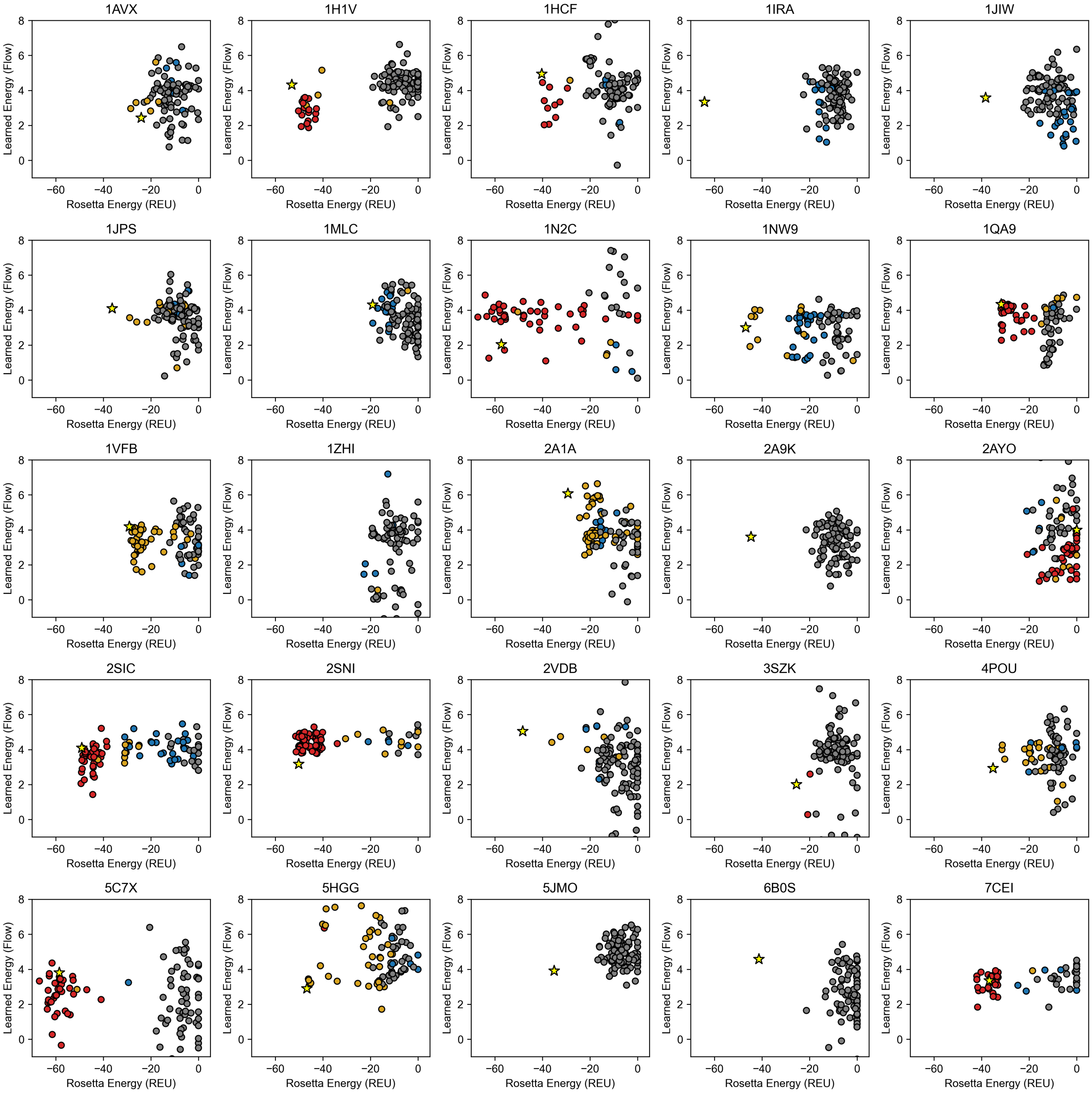

FIG. S7. Learned energy computed from integrating over flow trajectories, plotted against Rosetta energy of 120 docking poses generated from DFMDock for 25 targets in the DB5.5 dataset. Individual points are colored by their docking quality based on the CAPRI classification (incorrect: gray, acceptable: blue, medium: gold, high: red). Energy of the ground truth structure is shown as a yellow star.

bioRxiv preprint doi: https://doi.org/10.1101/2025.11.28.690021; this version posted December 17, 2025. The copyright holder for this preprint (which was not certified by peer review) is the author/funder, who has granted bioRxiv a license to display the preprint in perpetuity. It is made available under aCC-BY 4.0 International license.

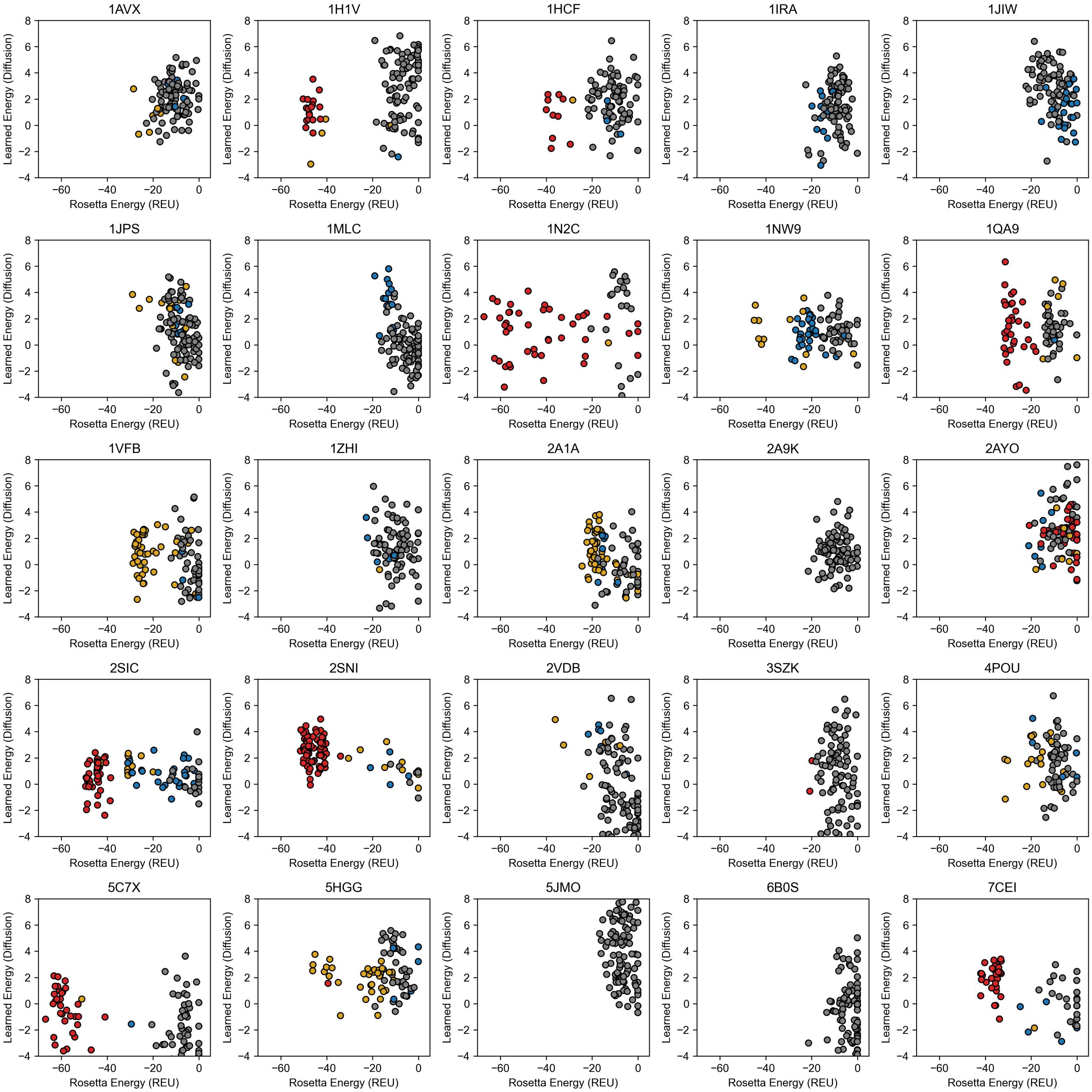

FIG. S8. Learned energy computed from integrating over flow trajectories, plotted against Rosetta energy of 120 docking poses generated from DFMDock for 25 targets in the DB5.5 dataset. Individual points are colored by their docking quality based on the CAPRI classification (incorrect: gray, acceptable: blue, medium: gold, high: red).

bioRxiv preprint doi: https://doi.org/10.1101/2025.11.28.690021; this version posted December 17, 2025. The copyright holder for this preprint (which was not certified by peer review) is the author/funder, who has granted bioRxiv a license to display the preprint in perpetuity. It is made available under aCC-BY 4.0 International license.

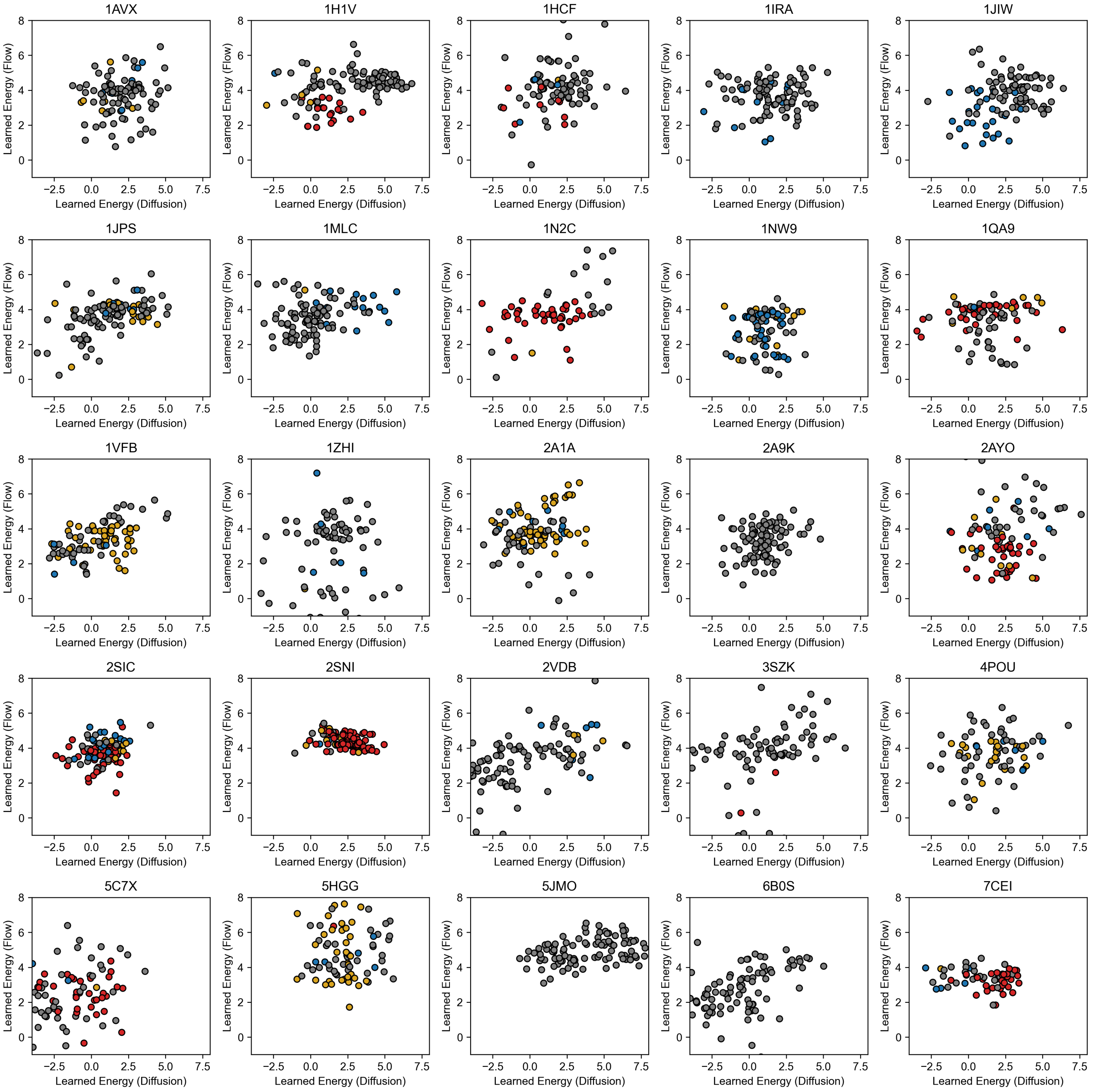

FIG. S9. Learned energy computed from integrating over flow trajectories plotted against integrating over diffusion trajectories, generated from DFMDock for 25 targets in the DB5.5 dataset. Individual points are colored by their docking quality based on the CAPRI classification (incorrect: gray, acceptable: blue, medium: gold, high: red).
