# Supplementary material for: Can We Extract Physics-like Energies from Generative Protein Diffusion Models?
Source: bioRxiv. 2025 Nov 29:2025.11.28.690021. Preprint. [Version 1] doi: 10.1101/2025.11.28.690021 (PMC12697549; doi:10.1101/2025.11.28.690021)
Supplement: Supplementary file 1 [file NIHPP2025.11.28.690021V1-supplement-1.pdf]

bioRxiv preprint doi: https://doi.org/10.1101/2025.11.28.690021; this version posted November 29, 2025. The copyright holder for this preprint (which was not certified by peer review) is the author/funder, who has granted bioRxiv a license to display the preprint in perpetuity. It is made available under aCC-BY 4.0 International license.

SUPPORTING INFORMATION

A. Dimensional Analysis of Key Equations

Each equation term below is analyzed for dimensionality, where $[M]$ represents units of mass, $[L]$ units of length, and $[T]$ units of (diffusion) time.

Equation (3):

$$dx_t = f(x_t, t)dt + g(t)dw_t$$

$$\begin{aligned} dx_t &\sim [L], \\ dt &\sim [T], \\ dw_t &\sim [T]^{1/2}, \\ f(x_t, t) &\sim \frac{[L]}{[T]}, \\ g(t) &\sim \frac{[L]}{[T]^{1/2}} \end{aligned}$$

Equation (4):

$$dx_t = \left[f(x_t, t) - g(t)^2 \nabla_x \log p(x_t, t)\right] dt + g(t)dw_t$$

$$\begin{aligned} g(t)^2 &\sim \frac{[L]^2}{[T]}, \\ \nabla_x \log p(x_t, t) &\sim \frac{1}{[L]}, \end{aligned}$$

Equations (10) and (11):

$$\begin{aligned} p(x_t, t) &= \frac{e^{-\beta E_t(x_t)}}{Z} \\ \log p(x_t, t) &= -\beta E_t(x_t) - \log Z \end{aligned}$$

$$\begin{aligned} &p(x_t, t) \sim [1], \\ &\beta E_t(x_t) \sim \frac{[T]^2}{[M][L]^2} \cdot \frac{[M][L]^2}{[T]^2} = [1], \\ &Z \sim [1] \end{aligned}$$

Equation (12):

$$\nabla_x \log p(x_t, t) = -\beta \nabla_x E_t(x_t)$$

$$\begin{aligned} &\nabla_x \log p(x_t, t) \sim \frac{[1]}{[L]}, \\ &\beta \nabla_x E_t(x_t) \sim \frac{[T]^2}{[M][L]^2} \cdot \frac{[M][L]}{[T]^2} = \frac{[1]}{[L]} \end{aligned}$$

Equation (14):

$$\Delta E = -\int F_t(x_t) \cdot dx_t$$

$$\begin{aligned} \Delta E &\sim \frac{[M][L]^2}{[T]^2}, \\ F_t(x_t) \cdot dx_t &\sim \frac{[M][L]^2}{[T]^2} \end{aligned}$$

Equation (19):

$$\Delta \log p = \int \left(\nabla_x \log p(x_t, t) \cdot \frac{\partial x}{\partial t} + \frac{\partial \log p(x_t, t)}{\partial t} \right) dt$$

$$\begin{aligned} &\Delta \log p \sim [1], \\ &\nabla_x \log p(x_t, t) \cdot \frac{\partial x}{\partial t} dt \\ &\quad \sim \frac{[1]}{[L]} \cdot \frac{[L]}{[T]} \cdot [T] = [1], \\ &\frac{\partial \log p(x_t, t)}{\partial t} dt \sim \frac{[1]}{[T]} \cdot [T] = [1] \end{aligned}$$

Equation (20):

$$\begin{aligned} \frac{\partial \log p(x_t, t)}{\partial t} &= \frac{1}{2} g(t)^2 \nabla_x \cdot \nabla_x \log p(x_t, t) \\ &\quad + \frac{1}{2} g(t)^2 \|\nabla_x \log p(x_t, t)\|^2 \end{aligned}$$

$$\begin{aligned} &\frac{\partial \log p(x_t, t)}{\partial t} \sim \frac{[1]}{[T]} \\ &\frac{1}{2} g(t)^2 \nabla_x \cdot \nabla_x \log p(x_t, t) \\ &\quad \sim \frac{[L]^2}{[T]} \cdot \frac{[1]}{[L]^2} = \frac{[1]}{[T]} \\ &\frac{1}{2} g(t)^2 \|\nabla_x \log p(x_t, t)\|^2 \\ &\quad \sim \frac{[L]^2}{[T]} \cdot \frac{[1]}{[L]^2} = \frac{[1]}{[T]} \end{aligned}$$

Equation (28):

$$\log p_0(x_0) = \log p_1(x_1) - \int_0^1 \frac{1}{2} g(t)^2 (\nabla_x \cdot \nabla_x \log p(x_t, t)) dt$$

$$\begin{aligned} &\log p_0(x_0) \sim [1], \\ &\log p10(x_1) \sim [1], \\ &\frac{1}{2} g(t)^2 (\nabla_x \cdot \nabla_x \log p(x_t, t)) dt \\ &\quad \sim \frac{[L]^2}{[T]} \cdot \frac{[1]}{[L]^2} \cdot [T] = [1] \end{aligned}$$

bioRxiv preprint doi: https://doi.org/10.1101/2025.11.28.690021; this version posted November 29, 2025. The copyright holder for this preprint (which was not certified by peer review) is the author/funder, who has granted bioRxiv a license to display the preprint in perpetuity. It is made available under aCC-BY 4.0 International license.

B. Derivation of Logarithmic Fokker Planck Equation

The Fokker-Planck equation is a PDE which describes the time-evolution of the marginal probability distribution $p(x_t, t)$ associated with a stochastic differential equation. It has many variants depending on the form of the SDE; for $dx_t = f(x_t, t)dt + g(t)dw_t$:

$$\begin{aligned}\frac{\partial p(x_t,t)}{\partial t} &= -\sum_{i=1}^{n} \frac{\partial}{\partial [x_t]_i} \left([f(x_t,t)]_i \; p(x_t,t)\right) \\ &+ \frac{1}{2} g(t)^2 \sum_{i=1}^{n} \frac{\partial^2}{\partial [x_t]_i^2} \left(p(x_t,t)\right)\end{aligned} \tag{S-1}$$

where n is the dimensionality of x_t, $[x_t]_i$ is the i-th component of x_t, and $[f(x_t,t)]_i$ is the i-th component of the drift term $f(x_t,t)$.

The Fokker-Planck equation is useful, but it is dependent on the value and spatial gradients of probability $p(x_t,t)$, neither of which we have; rather, our score model is trained to predict the spatial gradients of the *log* probability. We can convert between the true using the chain rule:

$$\begin{aligned}\frac{\partial \log f(x)}{\partial x_i} &= \log' f(x) \frac{\partial f(x)}{\partial x_i} \\ &= \frac{\partial f(x)}{\partial x_i} \frac{1}{|f(x)|}\end{aligned} \tag{S-2}$$

Since $p(x_t,t) > 0$,

$$\frac{\partial \log p(x_t,t)}{\partial t} = \frac{\partial p(x_t,t)}{\partial t} \frac{1}{p(x_t,t)} \tag{S-3}$$

So we can write the logarithmic Fokker-Planck equation as:

$$\frac{\partial \log p(x_t,t)}{\partial t} = \frac{\partial p(x_t,t)}{\partial t} \frac{1}{p(x_t,t)} \tag{S-4}$$

$$\begin{aligned}&= -\frac{1}{p(x_t,t)} \sum_{i=1}^{n} \frac{\partial}{\partial [x_t]_i} \left([f(x_t,t)]_i \; p(x_t,t)\right) \\ &+ \frac{1}{p(x_t,t)} \frac{1}{2} g(t)^2 \sum_{i=1}^{n} \frac{\partial^2}{\partial [x_t]_i^2} \left(p(x_t,t)\right)\end{aligned} \tag{S-5}$$

It's still dependent on derivatives of $p(x_t,t)$, but there's hope of converting them into logarithmic gradients as well by multiplying in $\frac{1}{p(x_t,t)}$. Expanding out the first-order term is easy:

$$\begin{aligned}\frac{\partial \log p(x_t,t)}{\partial t} &= -\frac{1}{p(x_t,t)} \sum_{i=1}^{n} \frac{\partial}{\partial [x_t]_i} \left([f(x_t,t)]_i\right) \; p(x_t,t) \\ &- \frac{1}{p(x_t,t)} \sum_{i=1}^{n} [f(x_t,t)]_i \; \frac{\partial}{\partial [x_t]_i} \left(p(x_t,t)\right) \\ &+ \frac{1}{p(x_t,t)} \frac{1}{2} g(t)^2 \sum_{i=1}^{n} \frac{\partial^2}{\partial [x_t]_i^2} \left(p(x_t,t)\right)\end{aligned} \tag{S-6}$$

The first $p(x_t,t)$ cancels, and the second becomes logarithmic due to the identity:

$$\begin{aligned}\frac{\partial \log p(x_t,t)}{\partial t} &= -\sum_{i=1}^{n} \frac{\partial}{\partial [x_t]_i} \left([f(x_t,t)]_i\right) \\ &- \sum_{i=1}^{n} [f(x_t,t)]_i \; \frac{\partial}{\partial [x_t]_i} \left(\log p(x_t,t)\right) \\ &+ \frac{1}{p(x_t,t)} \frac{1}{2} g(t)^2 \sum_{i=1}^{n} \frac{\partial^2}{\partial [x_t]_i^2} \left(p(x_t,t)\right)\end{aligned} \tag{S-7}$$

bioRxiv preprint doi: https://doi.org/10.1101/2025.11.28.690021; this version posted November 29, 2025. The copyright holder for this preprint (which was not certified by peer review) is the author/funder, who has granted bioRxiv a license to display the preprint in perpetuity. It is made available under aCC-BY 4.0 International license.

However, that second derivative in the third term will take a little more work. We can derive a similar identity to our first-order case:

$$\begin{aligned}\frac{\partial^2}{\partial[x_t]_i^2}p(x_t,t) &= \frac{\partial}{\partial[x_t]_i}\left(\frac{\partial}{\partial[x_t]_i}p(x_t,t)\right)\\ &= \frac{\partial}{\partial[x_t]_i}\left(\left(\frac{\partial}{\partial[x_t]_i}\log p(x_t,t)\right)\ p(x_t,t)\right)\\ &= \left(\frac{\partial^2}{\partial[x_t]_i^2}\log p(x_t,t)\right)p(x_t,t) + \left(\frac{\partial}{\partial[x_t]_i}\log p(x_t,t)\right)\left(\frac{\partial}{\partial[x_t]_i}p(x_t,t)\right)\\ &= \left(\frac{\partial^2}{\partial[x_t]_i^2}\log p(x_t,t)\right)p(x_t,t) + \left(\frac{\partial}{\partial[x_t]_i}\log p(x_t,t)\right)\left(\frac{\partial}{\partial[x_t]_i}\log p(x_t,t)\right)p(x_t,t)\\ &= \left(\frac{\partial^2}{\partial[x_t]_i^2}\log p(x_t,t)\right)p(x_t,t) + \left(\frac{\partial}{\partial[x_t]_i}\log p(x_t,t)\right)^2 p(x_t,t) \end{aligned} \tag{S-8}$$

Substituting this back into equation (S-7), the term splits into a second order term and a squared first order term, and the final remaining $p(x_t, t)$ cancels:

$$\begin{aligned}\frac{\partial \log p(x_t,t)}{\partial t} &= -\sum_{i=1}^{n}\frac{\partial}{\partial[x_t]_i}\left([f(x_t,t)]_i\right)\\ &\quad -\sum_{i=1}^{n}[f(x_t,t)]_i\,\frac{\partial}{\partial[x_t]_i}\left(\log p(x_t,t)\right)\\ &\quad +\frac{1}{2}g(t)^2\sum_{i=1}^{n}\frac{\partial^2}{\partial[x_t]_i^2}\log p(x_t,t)\\ &\quad +\frac{1}{2}g(t)^2\sum_{i=1}^{n}\left(\frac{\partial}{\partial[x_t]_i}\log p(x_t,t)\right)^2 \end{aligned} \tag{S-9}$$

giving us a form of the logarithmic Fokker-Planck equation which we can evaluate using only a trained score model. These sums can also be written using vector notation:

$$\begin{aligned}\frac{\partial \log p(x_t,t)}{\partial t} &= -\nabla_x\cdot f(x_t,t)\\ &\quad - f(x_t,t)\cdot\nabla_x\log p(x_t,t)\\ &\quad +\frac{1}{2}g(t)^2\nabla_x\cdot\nabla_x\log p(x_t,t)\\ &\quad +\frac{1}{2}g(t)^2\|\nabla_x\log p(x_t,t)\|^2 \end{aligned} \tag{S-10}$$

Finally, since our models are variance-exploding, their drift term $f(x_t, t) = 0$, meaning this simplifies to:

$$\begin{aligned}\frac{\partial \log p(x_t,t)}{\partial t} &= \frac{1}{2}g(t)^2\nabla_x\cdot\nabla_x\log p(x_t,t)\\ &\quad +\frac{1}{2}g(t)^2\|\nabla_x\log p(x_t,t)\|^2 \end{aligned} \tag{20}$$

Technically, the process whose marginal distribution we care about is the *reverse* diffusion process, whose drift term is not zero. As mentioned above, the reverse diffusion process is chosen precisely so that its marginal distribution matches that of the forward process, and therefore the Fokker-Planck equations should be identical for both (in fact, [39] derives the reverse SDE precisely by matching the Fokker-Planck for the forward and backward equations). For thoroughness, we will also derive the marginal distribution for the reverse process.

B.1 Reverse-Time Fokker-Planck

The most important thing to keep track of is the direction of time and the sign of its change. Importantly, the Fokker-Planck assumes a positive dt and a forward-time wiener process dw_t, but the reverse SDE involves a negative

bioRxiv preprint doi: https://doi.org/10.1101/2025.11.28.690021; this version posted November 29, 2025. The copyright holder for this preprint (which was not certified by peer review) is the author/funder, who has granted bioRxiv a license to display the preprint in perpetuity. It is made available under aCC-BY 4.0 International license.

dt and a reverse-time wiener process $d\overline{w}_t$. To rectify this, we use $s = 1 - t$ in place of t; then eq. (4) becomes:

$$dx_s = [-f(x_s, s) + g(s)^2 \nabla_x \log p(x_s, s)]ds + g(s)dw_s \tag{S-11}$$

The logarithmic Fokker-Planck equation for this SDE is then:

$$\begin{aligned}
\frac{\partial \log p(x_s, s)}{\partial s} &= -\nabla_x \cdot [-f(x_s, s) + g(s)^2 \nabla_x \log p(x_s, s)] \\
&- [-f(x_s, s) + g(s)^2 \nabla_x \log p(x_s, s)] \cdot \nabla_x \log p(x_s, s) \\
&+ \frac{1}{2} g(s)^2 \nabla_x \cdot \nabla_x \log p(x_s, s) \\
&+ \frac{1}{2} g(s)^2 \|\nabla_x \log p(x_s, s)\|^2 \\
\\
&= \nabla_x \cdot f(x_s, s) - g(s)^2 \nabla_x \cdot \nabla_x \log p(x_s, s) \\
&+ f(x_s, s) \cdot \nabla_x \log p(x_s, s) - g(s)^2 \nabla_x \log p(x_s, s) \cdot \nabla_x \log p(x_s, s) \\
&+ \frac{1}{2} g(s)^2 \nabla_x \cdot \nabla_x \log p(x_s, s) \\
&+ \frac{1}{2} g(s)^2 \|\nabla_x \log p(x_s, s)\|^2 \\
\\
&= \nabla_x \cdot f(x_s, s) \\
&+ f(x_s, s) \cdot \nabla_x \log p(x_s, s) \\
&- \frac{1}{2} g(s)^2 \nabla_x \cdot \nabla_x \log p(x_s, s) \\
&- \frac{1}{2} g(s)^2 \|\nabla_x \log p(x_s, s)\|^2
\end{aligned} \tag{S-12}$$

Changing variables back to t, the chain rule means we swap the sign of the RHS, yielding precisely our original equation:

$$\frac{\partial \log p(x_t, t)}{\partial t} = \frac{\partial \log p(x_s, s)}{\partial s} \cdot \frac{\partial s}{\partial t} \tag{S-13}$$

$$\begin{aligned}
&= -\nabla_x \cdot f(x_t, t) \\
&- f(x_t, t) \cdot \nabla_x \log p(x_t, t) \\
&+ \frac{1}{2} g(t)^2 \nabla_x \cdot \nabla_x \log p(x_t, t) \\
&+ \frac{1}{2} g(t)^2 \|\nabla_x \log p(x_t, t)\|^2
\end{aligned} \tag{S-14}$$

To save on this algebraic manipulation, we always used the Fokker-Planck of the forward equation in the main body of the paper, assuming $dt > 0$ and integrating t from $0 \to 1$.

C. Derivation of Flow Trajectory Integral

When applying eq. (25) to a trajectory with a well-defined $\frac{dx_t}{dt}$, like an ODE, we can further simplify the integral using the definition. In the case of the flow trajectory, eq. (27), this leads to a cancellation between two of the three terms, leaving only the divergence term behind. Starting with the dx term of eq. (19):

$$\begin{aligned}
\nabla_x \log p(x_t, t) \cdot \frac{dx_t}{dt} &= \nabla_x \log p(x_t, t) \cdot \left(-\frac{1}{2} g(t)^2 \nabla_x \log p(x_t, t) \right) \\
&= -\frac{1}{2} g(t)^2 \left(\nabla_x \log p(x_t, t) \cdot \nabla_x \log p(x_t, t) \right) \\
&= -\frac{1}{2} g(t)^2 \|\nabla_x \log p(x_t, t)\|^2
\end{aligned} \tag{S-15}$$

bioRxiv preprint doi: https://doi.org/10.1101/2025.11.28.690021; this version posted November 29, 2025. The copyright holder for this preprint (which was not certified by peer review) is the author/funder, who has granted bioRxiv a license to display the preprint in perpetuity. It is made available under aCC-BY 4.0 International license.

Then approximating $\nabla_x \log p(x_t, t)$ with $s_\theta(x_t, t)$ and plugging into eq. (25):

$$
\begin{aligned}
\log p_0(x_0) &= \log p_1(x_1) \\
&- \int_0^1 \left(s_\theta(x_t, t) \cdot \frac{dx_t}{dt} + \frac{1}{2} g(t)^2 \mathrm{Tr}(\nabla_x s_\theta(x_t, t)) + \frac{1}{2} g(t)^2 \|s_\theta(x_t, t)\|^2 \right) dt \qquad (25) \\
&= \log p_1(x_1) \\
&- \int_0^1 \left(-\cancel{\frac{1}{2} g(t)^2 \|s_\theta(x_t, t)\|^2} + \frac{1}{2} g(t)^2 \mathrm{Tr}(\nabla_x s_\theta(x_t, t)) + \cancel{\frac{1}{2} g(t)^2 \|s_\theta(x_t, t)\|^2} \right) dt \\
&= \log p_1(x_1) - \int_0^1 \frac{1}{2} g(t)^2 \mathrm{Tr}(\nabla_x s_\theta(x_t, t)) dt \qquad (28)
\end{aligned}
$$

bioRxiv preprint doi: https://doi.org/10.1101/2025.11.28.690021; this version posted November 29, 2025. The copyright holder for this preprint (which was not certified by peer review) is the author/funder, who has granted bioRxiv a license to display the preprint in perpetuity. It is made available under aCC-BY 4.0 International license.

D. Pseudocodes for log likelihood integrals

These are pseudocode algorithms for integrating over discrete diffusion trajectories using the trapezoid rule (Algorithm 1) and flow trajectories using black-box ODE solvers (Algorithm 2).

Algorithm 1 Trapezoidal Integration over Diffusion Trajectories

Input: Diffusion trajectory samples $\{(x_{t_0}, t_0), (x_{t_1}, t_1), \ldots, (x_{t_n}, t_n)\}$ where $t_i \in [0, 1], t_0 = 0, t_1 = 1$; score model $s_\theta(x_t, t)$; noise schedule $g(t)$
Output: Log-likelihood estimate $\log p_0(x_0) = \log p_1(x_1) - \Delta \log p$
function INTEGRAND(x_t, t,Δx, Δt)
  return $s_\theta(x_t, t) \cdot \Delta x + \frac{1}{2} g(t)^2 \mathrm{Tr}(\nabla_x s_\theta(x_t, t)) + \frac{1}{2} g(t)^2 \|s_\theta(x_t, t)\|^2$
end function
Initialize $\Delta \log p \leftarrow 0$
for $i = 0$ to $n - 1$ **do**
  $\Delta x_i \leftarrow x_{t_{i+1}} - x_{t_i}$
  $\Delta t_i \leftarrow t_{i+1} - t_i$
  $f_a \leftarrow \mathrm{Integrand}(x_{t_i}, t_i, \Delta x_i, \Delta t_i)$
  $f_b \leftarrow \mathrm{Integrand}(x_{t_{i+1}}, t_{i+1}, \Delta x_i, \Delta t_i)$
  $\Delta \log p \leftarrow \Delta \log p + \frac{1}{2}(f_a + f_b)$
end for
Compute $\log p_1(x_1) \sim \mathcal{N}(0, I)$ ▷ Gaussian prior
$\log p_0(x_0) \leftarrow \log p_1(x_1) - \Delta \log p$
return $\log p_0(x_0)$

Algorithm 2 ODE Integration over Flow Paths

Input: Diffusion sample x_0; score model $s_\theta(x_t, t)$; noise schedule $g(t)$
Output: Log-likelihood $\log p_0(x_0)$
function DRIFT(x_t, t)
  return $-g(t)^2 \cdot s_\theta(x_t, t)$
end function
function TRACEJACOBIAN(x_t, t)
  return $\frac{1}{2} g(t)^2 \mathrm{Tr}(\nabla_x s_\theta(x_t, t))$
end function
Solve the following ODEs from $t = 0$ to $t = 1$ using an ODE solver:

- $\frac{dx_t}{dt} = \mathrm{Drift}(x_t, t)$ with initial condition x_0
- $\frac{d(\mathtt{logp})}{dt} = \mathrm{TraceJacobian}(x_t, t)$ with initial value 0
- **return** $\{x_t|_{t=0}^{1}\}, \Delta \log p$

Compute $\log p_1(x_1) \sim \mathcal{N}(0, I)$ ▷ Gaussian prior
$\log p_0(x_0) \leftarrow \log p_1(x_1) - \Delta \log p$
return $\log p_0(x_0)$

bioRxiv preprint doi: https://doi.org/10.1101/2025.11.28.690021; this version posted November 29, 2025. The copyright holder for this preprint (which was not certified by peer review) is the author/funder, who has granted bioRxiv a license to display the preprint in perpetuity. It is made available under aCC-BY 4.0 International license.

E. Additional supplementary figures

Supp. Fig.	y-axis	x-axis
Supp. Fig. S1	Diffusion Trajectory Learned Energy	Interface RMSD
Supp. Fig. S2	Diffusion Trajectory Learned Energy	DockQ
Supp. Fig. S3	Flow Trajectory Learned Energy	Interface RMSD
Supp. Fig. S4	Flow Trajectory Learned Energy	DockQ
Supp. Fig. S5	Rosetta Energy	Interface RMSD
Supp. Fig. S6	Rosetta Energy	DockQ
Supp. Fig. S7	Flow Trajectory Learned Energy	Rosetta Energy
Supp. Fig. S8	Diffusion Trajectory Learned Energy	Rosetta Energy
Supp. Fig. S9	Diffusion Trajectory Learned Energy	Flow Trajectory Learned Energy

bioRxiv preprint doi: https://doi.org/10.1101/2025.11.28.690021; this version posted November 29, 2025. The copyright holder for this preprint (which was not certified by peer review) is the author/funder, who has granted bioRxiv a license to display the preprint in perpetuity. It is made available under aCC-BY 4.0 International license.

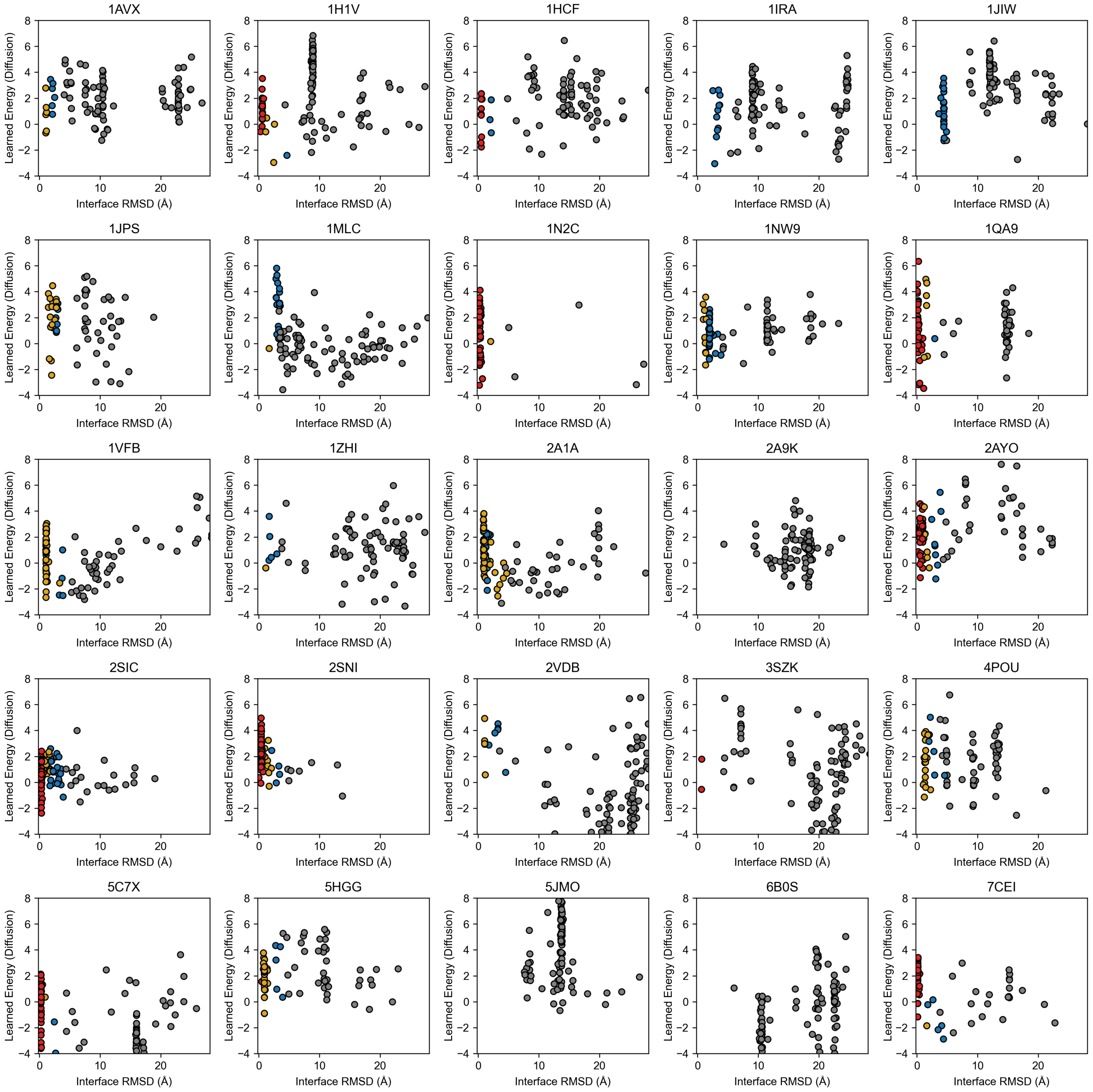

FIG. S1. Learned energy computed from integrating over diffusion trajectories, plotted against interface RMSD of 120 docking poses generated from DFMDock for 25 targets in the DB5.5 dataset. Individual points are colored by their docking quality based on the CAPRI classification (incorrect: gray, acceptable: blue, medium: gold, high: red).

bioRxiv preprint doi: https://doi.org/10.1101/2025.11.28.690021; this version posted November 29, 2025. The copyright holder for this preprint (which was not certified by peer review) is the author/funder, who has granted bioRxiv a license to display the preprint in perpetuity. It is made available under aCC-BY 4.0 International license.

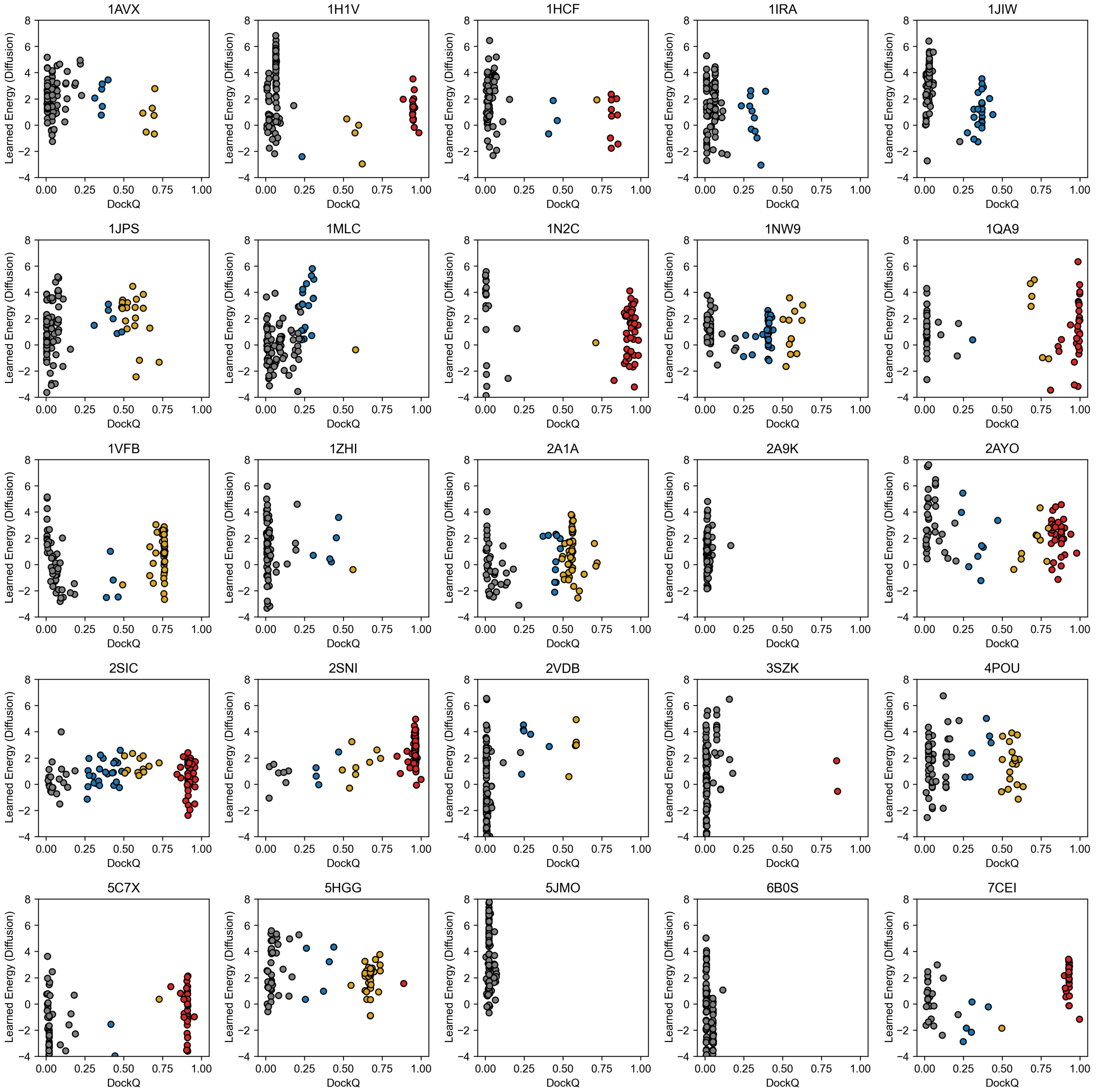

FIG. S2. Learned energy computed from integrating over diffusion trajectories, plotted against DockQ of 120 docking poses generated from DFMDock for 25 targets in the DB5.5 dataset. Individual points are colored by their docking quality based on the CAPRI classification (incorrect: gray, acceptable: blue, medium: gold, high: red).

bioRxiv preprint doi: https://doi.org/10.1101/2025.11.28.690021; this version posted November 29, 2025. The copyright holder for this preprint (which was not certified by peer review) is the author/funder, who has granted bioRxiv a license to display the preprint in perpetuity. It is made available under aCC-BY 4.0 International license.

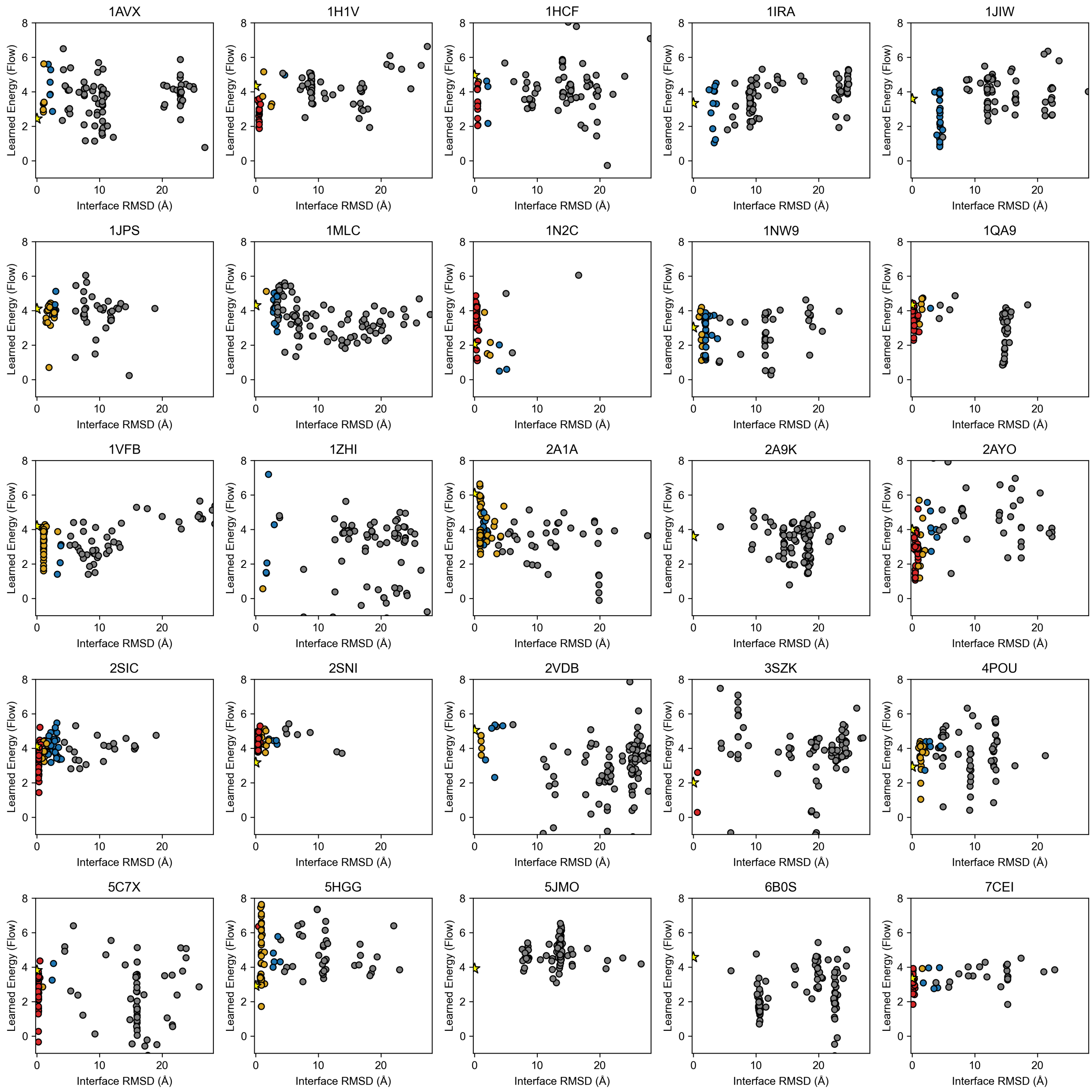

FIG. S3. Learned energy computed from integrating over flow trajectories, plotted against interface RMSD of 120 docking poses generated from DFMDock for 25 targets in the DB5.5 dataset. Individual points are colored by their docking quality based on the CAPRI classification (incorrect: gray, acceptable: blue, medium: gold, high: red). Energy of the ground truth structure is shown as a yellow star.

bioRxiv preprint doi: https://doi.org/10.1101/2025.11.28.690021; this version posted November 29, 2025. The copyright holder for this preprint (which was not certified by peer review) is the author/funder, who has granted bioRxiv a license to display the preprint in perpetuity. It is made available under aCC-BY 4.0 International license.

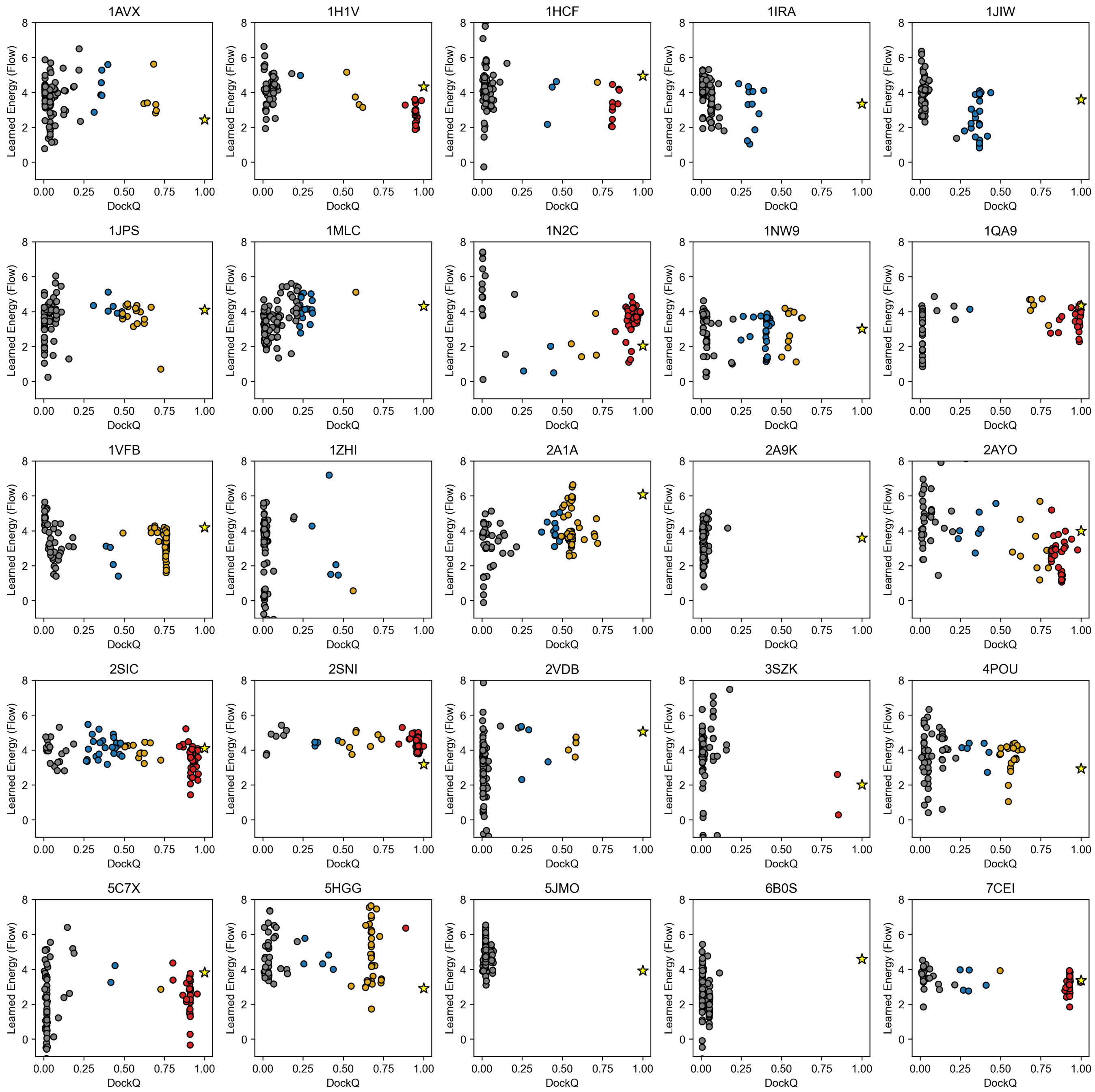

FIG. S4. Learned energy computed from integrating over flow trajectories, plotted against DockQ of 120 docking poses generated from DFMDock for 25 targets in the DB5.5 dataset. Individual points are colored by their docking quality based on the CAPRI classification (incorrect: gray, acceptable: blue, medium: gold, high: red). Energy of the ground truth structure is shown as a yellow star.

bioRxiv preprint doi: https://doi.org/10.1101/2025.11.28.690021; this version posted November 29, 2025. The copyright holder for this preprint (which was not certified by peer review) is the author/funder, who has granted bioRxiv a license to display the preprint in perpetuity. It is made available under aCC-BY 4.0 International license.

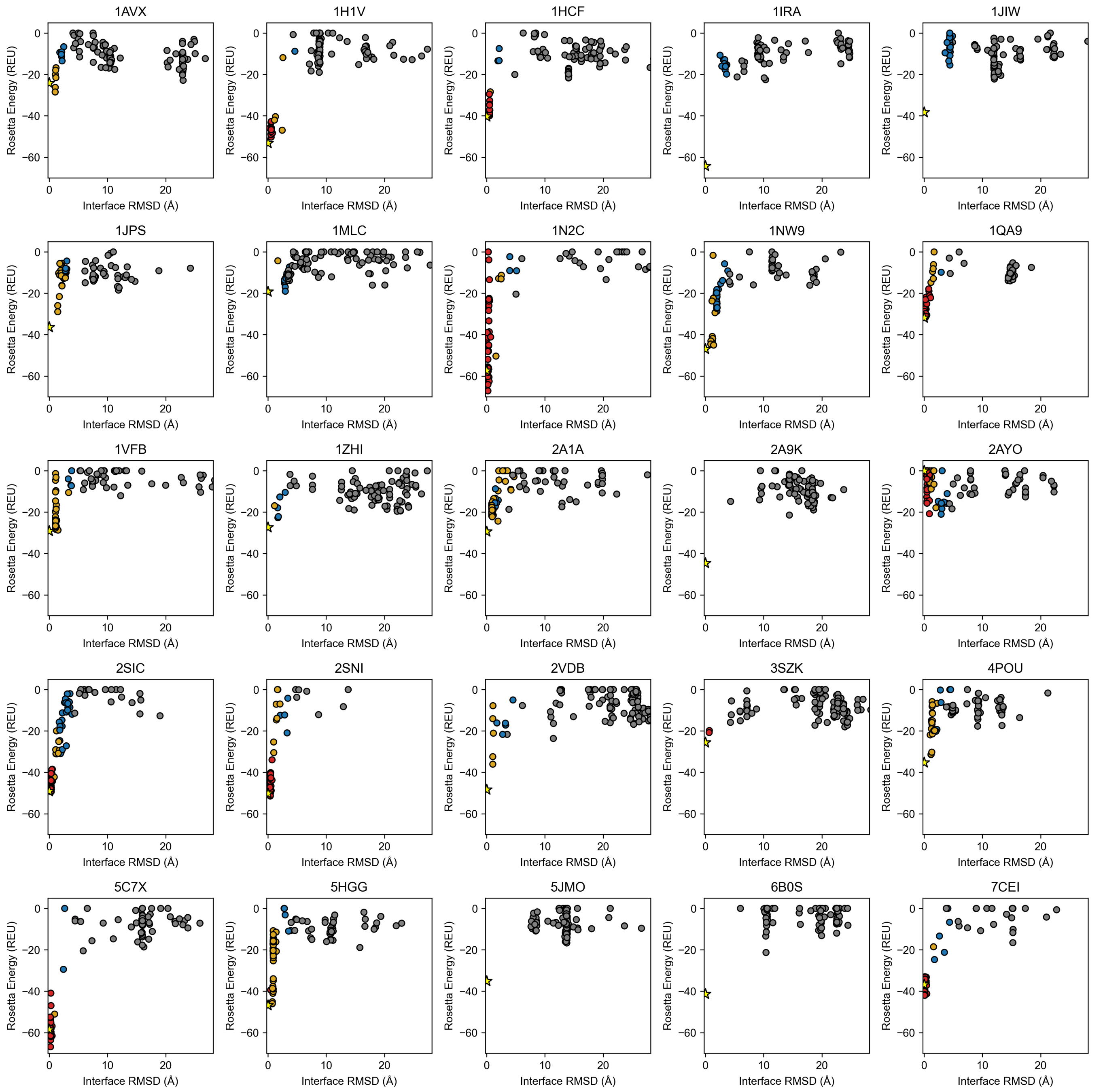

FIG. S5. Rosetta energy plotted against interface RMSD of 120 docking poses generated from DFMDock for 25 targets in the DB5.5 dataset. Individual points are colored by their docking quality based on the CAPRI classification (incorrect: gray, acceptable: blue, medium: gold, high: red). Energy of the ground truth structure is shown as a yellow star.

bioRxiv preprint doi: https://doi.org/10.1101/2025.11.28.690021; this version posted November 29, 2025. The copyright holder for this preprint (which was not certified by peer review) is the author/funder, who has granted bioRxiv a license to display the preprint in perpetuity. It is made available under aCC-BY 4.0 International license.

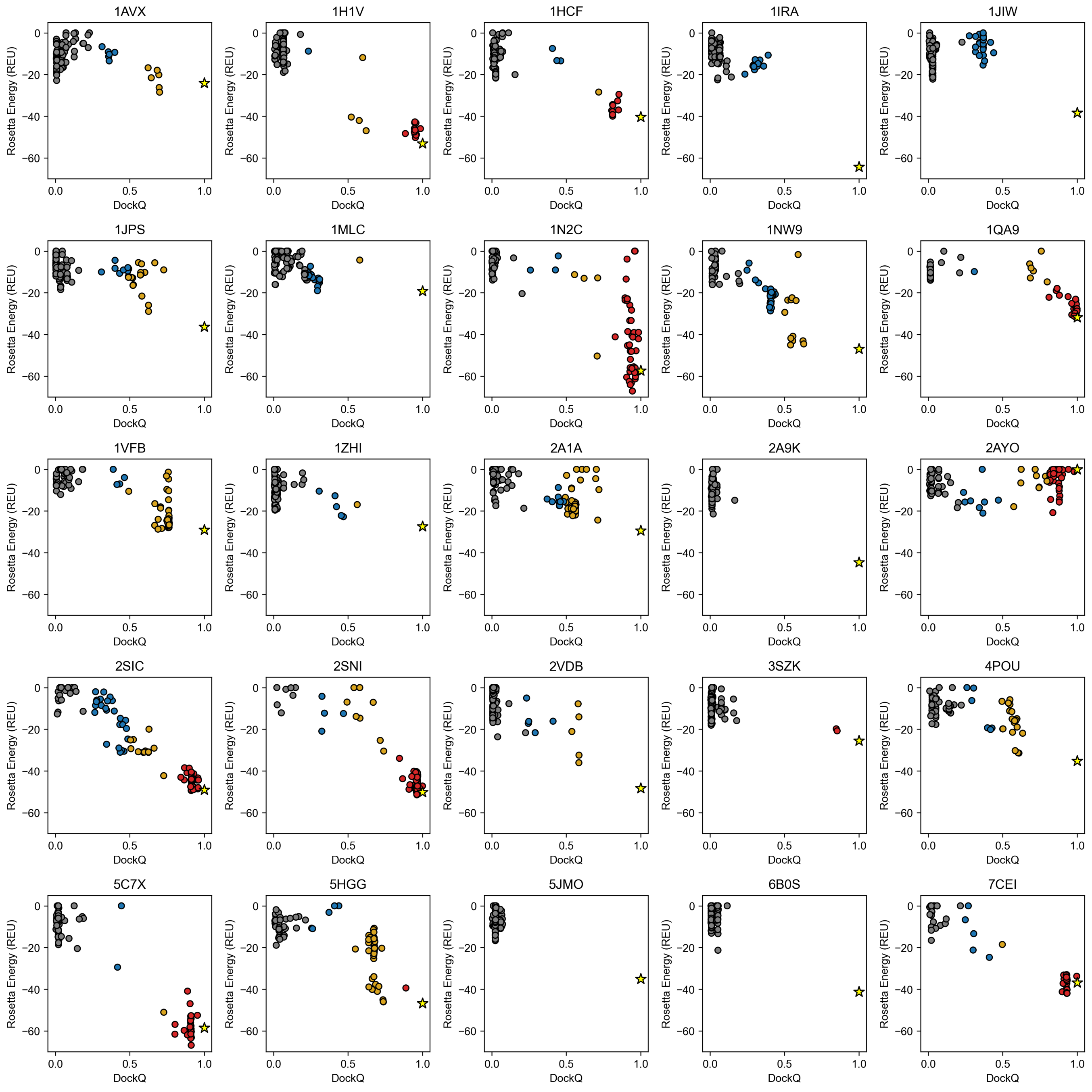

FIG. S6. Rosetta energy plotted against DockQ of 120 docking poses generated from DFMDock for 25 targets in the DB5.5 dataset. Individual points are colored by their docking quality based on the CAPRI classification (incorrect: gray, acceptable: blue, medium: gold, high: red). Energy of the ground truth structure is shown as a yellow star.

bioRxiv preprint doi: https://doi.org/10.1101/2025.11.28.690021; this version posted November 29, 2025. The copyright holder for this preprint (which was not certified by peer review) is the author/funder, who has granted bioRxiv a license to display the preprint in perpetuity. It is made available under aCC-BY 4.0 International license.

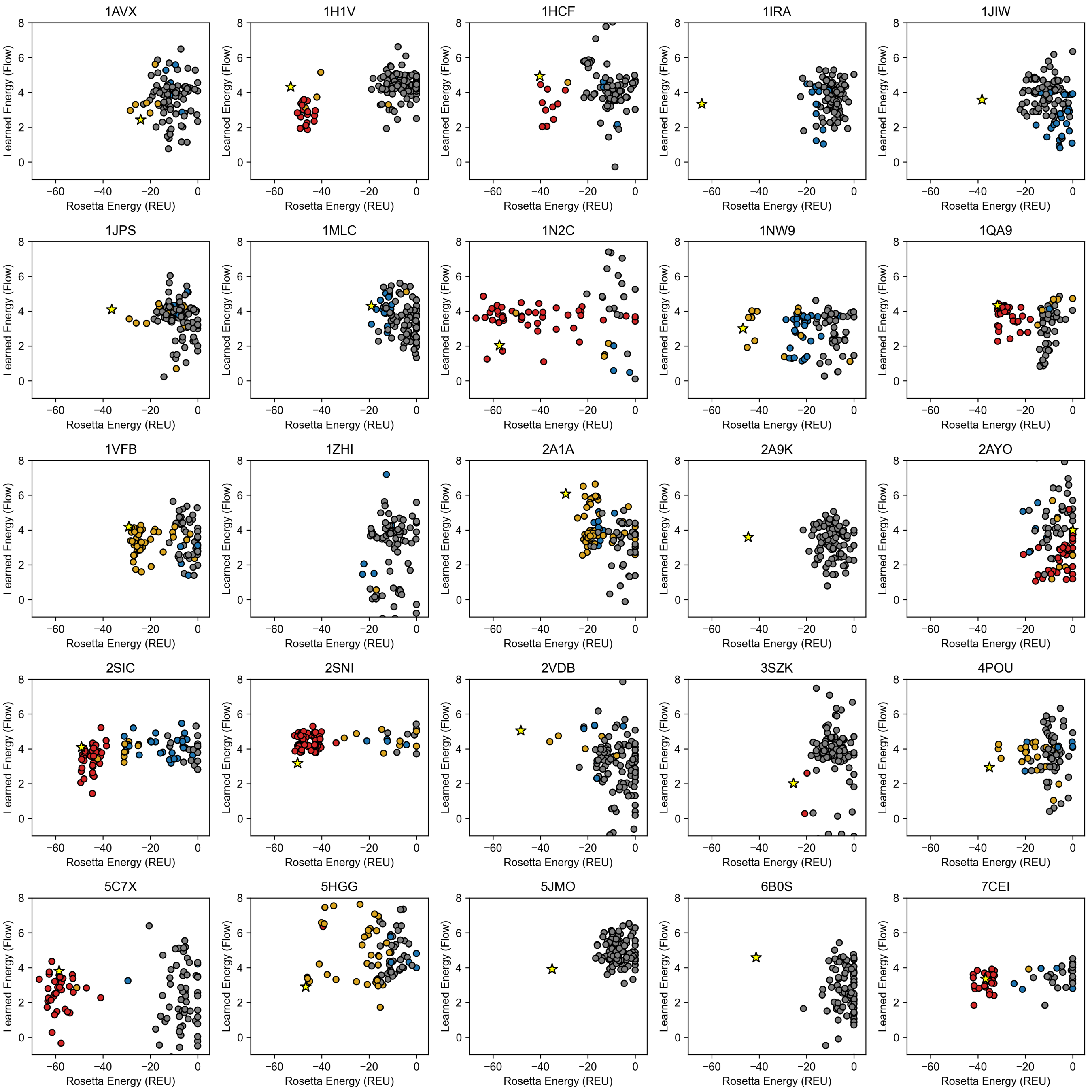

FIG. S7. Learned energy computed from integrating over flow trajectories, plotted against Rosetta energy of 120 docking poses generated from DFMDock for 25 targets in the DB5.5 dataset. Individual points are colored by their docking quality based on the CAPRI classification (incorrect: gray, acceptable: blue, medium: gold, high: red). Energy of the ground truth structure is shown as a yellow star.

bioRxiv preprint doi: https://doi.org/10.1101/2025.11.28.690021; this version posted November 29, 2025. The copyright holder for this preprint (which was not certified by peer review) is the author/funder, who has granted bioRxiv a license to display the preprint in perpetuity. It is made available under aCC-BY 4.0 International license.

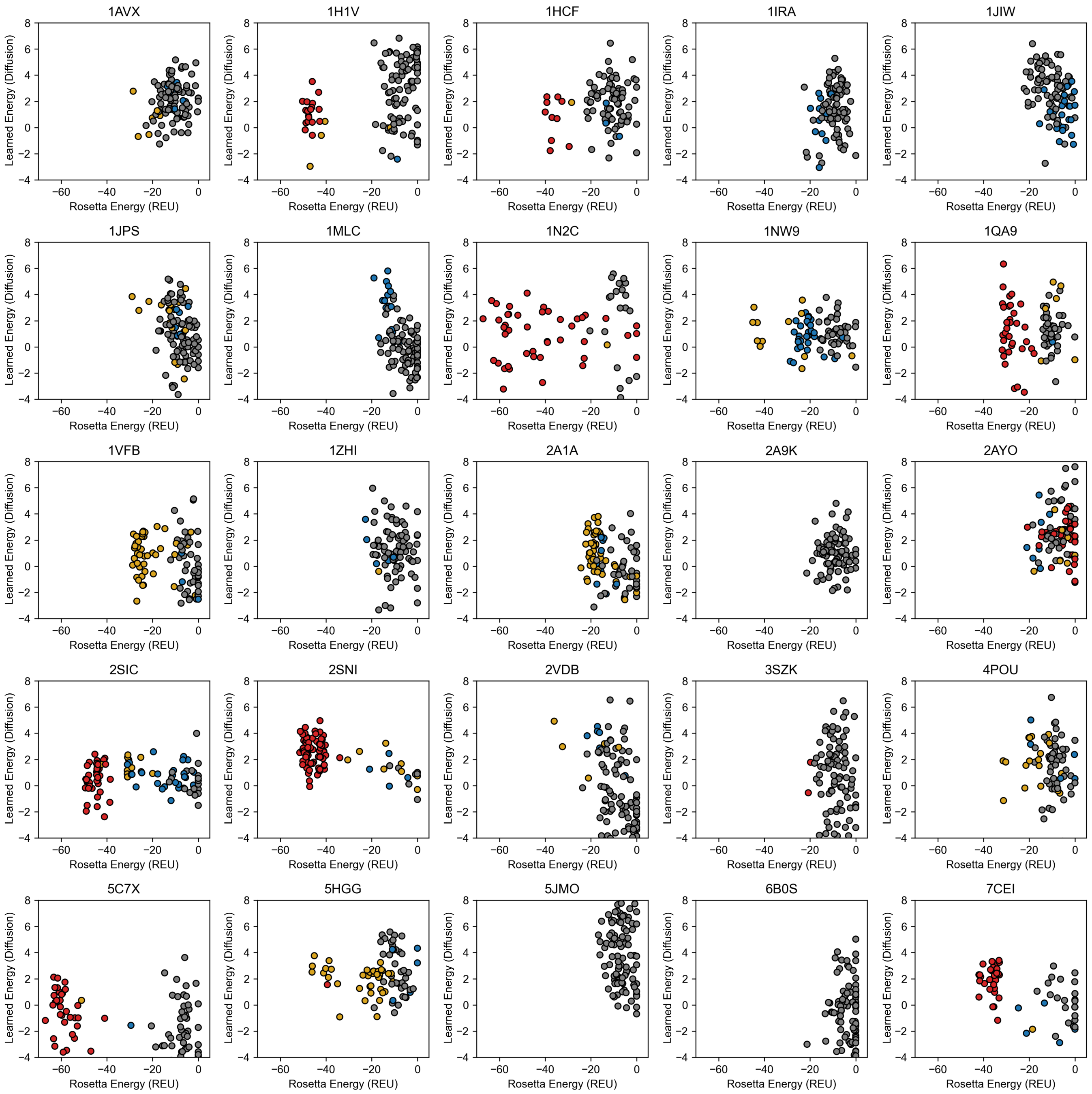

FIG. S8. Learned energy computed from integrating over flow trajectories, plotted against Rosetta energy of 120 docking poses generated from DFMDock for 25 targets in the DB5.5 dataset. Individual points are colored by their docking quality based on the CAPRI classification (incorrect: gray, acceptable: blue, medium: gold, high: red). Energy of the ground truth structure is shown as a yellow star.

bioRxiv preprint doi: https://doi.org/10.1101/2025.11.28.690021; this version posted November 29, 2025. The copyright holder for this preprint (which was not certified by peer review) is the author/funder, who has granted bioRxiv a license to display the preprint in perpetuity. It is made available under aCC-BY 4.0 International license.

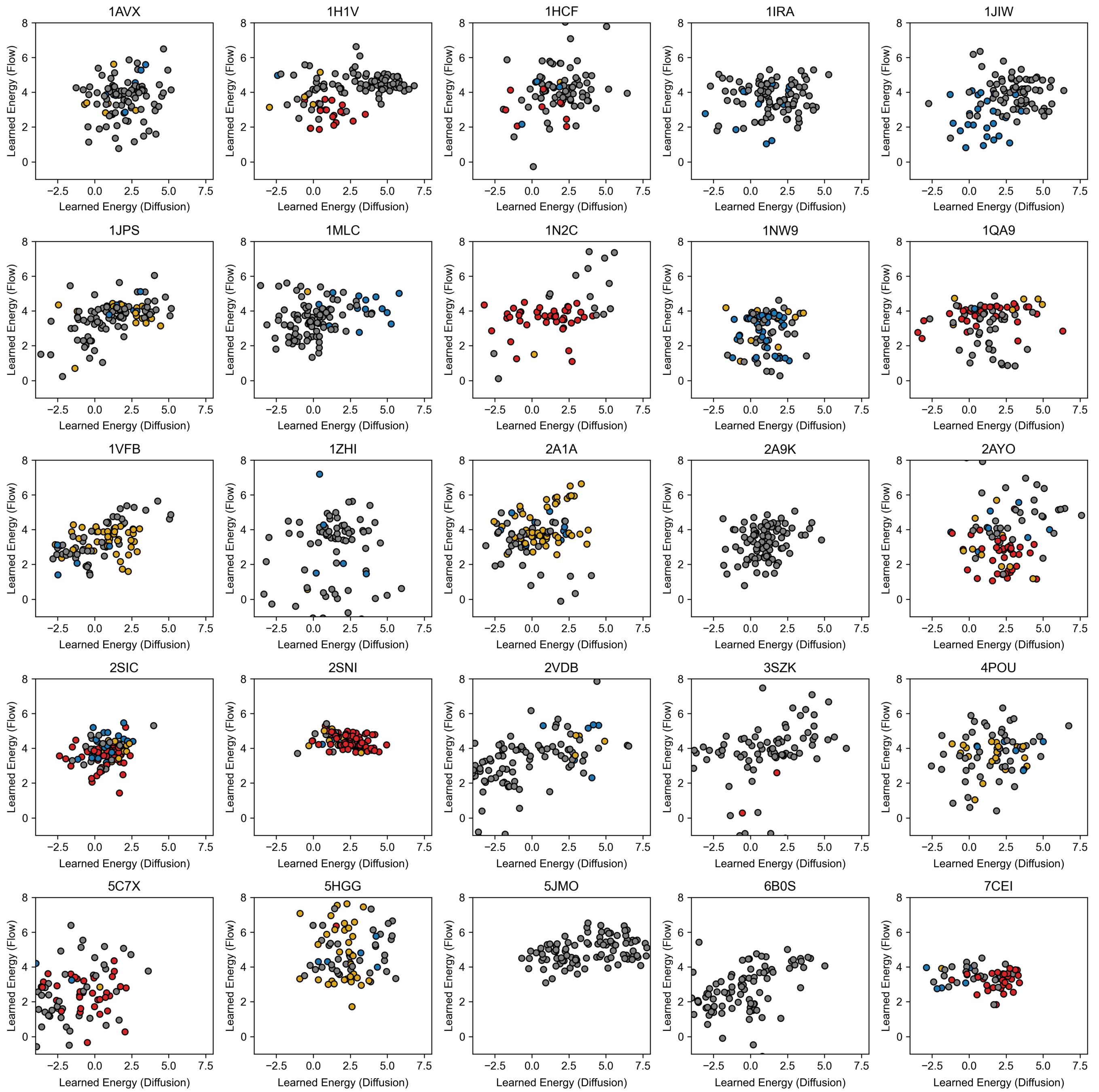

FIG. S9. Learned energy computed from integrating over flow trajectories plotted against integrating over diffusion trajectories, generated from DFMDock for 25 targets in the DB5.5 dataset. Individual points are colored by their docking quality based on the CAPRI classification (incorrect: gray, acceptable: blue, medium: gold, high: red). Energy of the ground truth structure is shown as a yellow star.
